# Dynamics of the Gene Regulatory Network of HIV-1 and the Role of Viral Non-coding RNAs on Latency Reversion

**DOI:** 10.3389/fphys.2018.01364

**Published:** 2018-09-28

**Authors:** Antonio Bensussen, Christian Torres-Sosa, Ramón A. Gonzalez, José Díaz

**Affiliations:** ^1^Laboratory of Gene Networks Dynamics, Centro de Investigación en Dinámica Celular, Universidad Autónoma del Estado de Morelos, Cuernavaca, Mexico; ^2^Centro de Ciencias de la Complejidad, Universidad Nacional Autónoma de México, Ciudad de México, Mexico; ^3^Instituto de Ciencias Físicas, Universidad Nacional Autónoma de México, Cuernavaca, Mexico; ^4^Laboratory of Molecular Virology, Centro de Investigación en Dinámica Celular, Universidad Autónoma del Estado de Morelos, Cuernavaca, Mexico

**Keywords:** HIV-1, viral non-coding RNAs, reservoirs, antiretroviral therapy, LRAs, dynamics, Boolean networks

## Abstract

The use of latency reversing agents (LRAs) is currently a promising approach to eliminate latent reservoirs of HIV-1. However, this strategy has not been successful *in vivo*. It has been proposed that cellular post-transcriptional mechanisms are implicated in the underperformance of LRAs, but it is not clear whether proviral regulatory elements like viral non-coding RNAs (vncRNAs) are also implicated. In order to visualize the complexity of the HIV-1 gene expression, we used experimental data to construct a gene regulatory network (GRN) of latent proviruses in resting CD4+ T cells. We then analyzed the dynamics of this GRN using Boolean and continuous mathematical models. Our simulations predict that vncRNAs are able to counteract the activity of LRAs, which may explain the failure of these compounds to reactivate latent reservoirs of HIV-1. Moreover, our results also predict that using inhibitors of histone methyltransferases, such as chaetocin, together with releasers of the positive transcription elongation factor (P-TEFb), like JQ1, may increase proviral reactivation despite self-repressive effects of vncRNAs.

## Introduction

Combined antiretroviral therapy (cART) is currently the most effective approach to control the chronic infection of HIV-1. However, cART does not eliminate the virus even with treatment intensification (Dinoso et al., 2009). This occurs because HIV-1 is able to form long-lived reservoirs by remaining latent within resting memory CD4+ T-cells (Siliciano et al., 2003; Siliciano and Greene, 2011; Cohn et al., 2015). Recently it has been proposed the use of LRAs in combination with cART to eliminate latently infected cells. Ideally this “*shock-and-kill*” strategy could purge viral reservoirs because when LRAs reactivate latently infected cells, those cells may be eliminated by self HIV-1 replication or by action of the immune system while cART prevents the formation of new viral reservoirs (Deeks, 2012). Despite many *in vitro* observations suggest that this strategy can be a promising approach (Deeks, 2012), clinical trials with LRAs have shown that it is ineffective *in vivo* (Bullen et al., 2014). Stochastic modeling of latently infected cells indicated that the clinical underperformance of LRAs is due to their inability to minimize the size of the viral reservoirs (Hill et al., 2014). Furthermore, this study suggested that LRAs must reduce the size of viral reservoirs 10,000-fold to prevent HIV rebounds after cART (Hill et al., 2014), an objective that cannot be reached with current treatments (Cillo et al., 2014).

The “*shock-and-kill*” strategy is based on the assumption that proviral reactivation depends only on the immunological activation of the infected cells. However, recent findings suggest that this assumption is not entirely true, since it has been observed that the provirus is able to autonomously regulate its latency using the positive feedback loop of trans-activator of transcription (Tat) independently of cell activation (Razooky et al., 2015). During early stages of infection, Tat is synthesized at low levels that fluctuate because of cell's downregulation of the provirus (Weinberger and Shenk, 2007). When these transcriptional fluctuations are sustained, the activity of Tat initiates a positive feedback loop which boosts proviral transcription by recruiting P-TEFb in order to increase the synthesis of full-length viral RNAs (Weinberger and Shenk, 2007; Romani et al., 2010). In a biological context the two classical functions of positive feedback loops are to amplify and to sustain gene expression (Zhang Q. et al., 2014), however the architecture of the Tat circuit only amplifies transcriptional fluctuations making the gene expression of provirus transitory (Weinberger et al., 2005; Weinberger and Shenk, 2007). This architecture constitutes a mechanism of negative self-regulation of HIV-1, which may hinder viral reactivation (Razooky et al., 2015), and therefore may obstruct the activity of LRAs. Nevertheless, Tat is not the only structural component of HIV-1 that has a regulatory circuit. It has been observed that several vncRNAs have their own positive and negative feedback loops that may increase or suppress gene expression of the provirus (Groen and Morris, 2013; Saayman et al., 2014; Zhang Y. et al., 2014; Suzuki et al., 2015). It has been suggested that those vncRNAs have a secondary role on latency maintaining (Suzuki et al., 2015) and it is not clear whether such viral components participate in the low efficiency of the LRAs.

**Figure 1:**
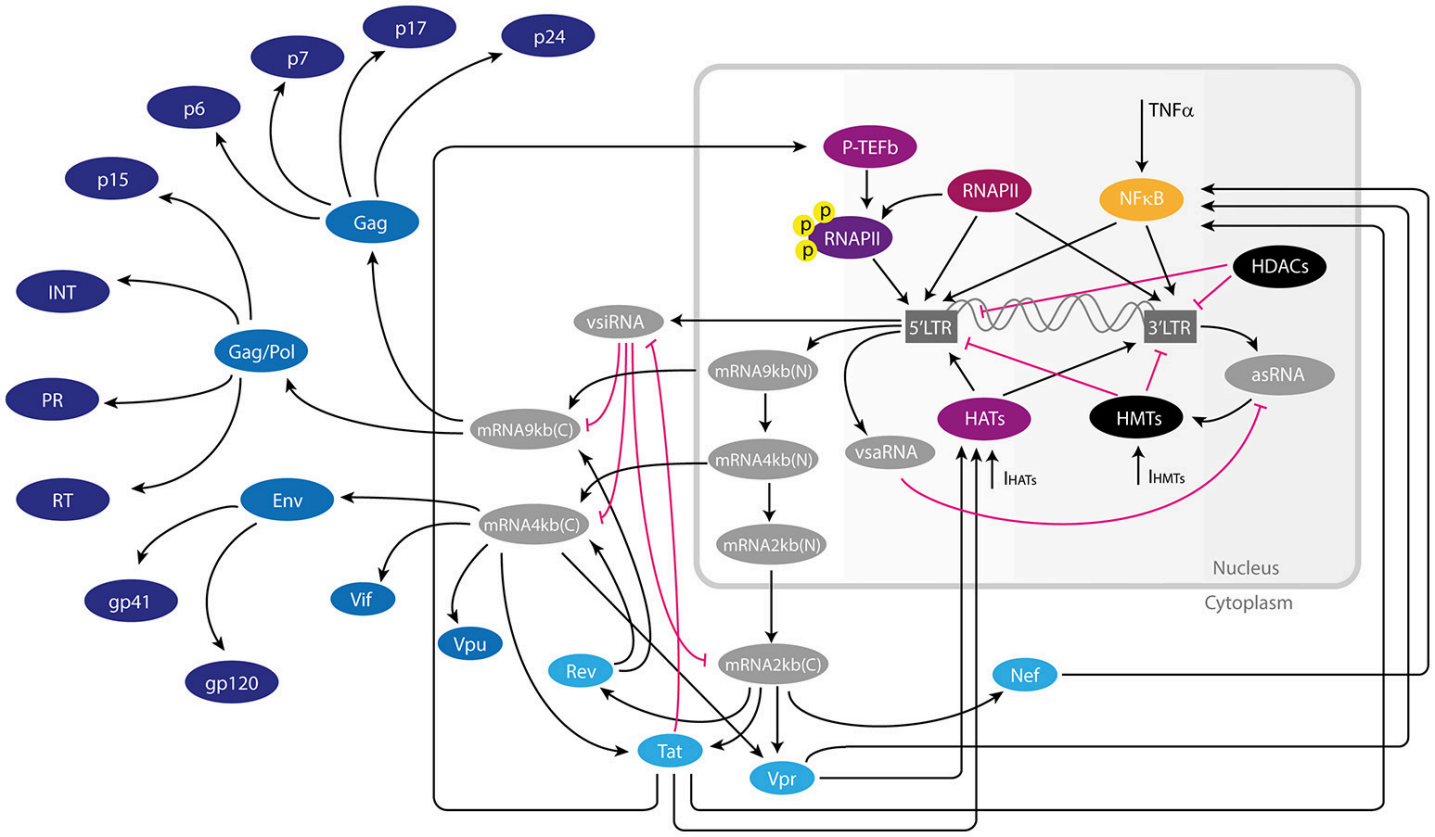
Gene regulatory network of HIV-1 provirus. Inside latently infected resting CD4+-T cells, the provirus can be activated by transcription factors and chromatin remodeling machinery of the host during immunological stimulation. When this occurs, the provirus expresses a wide variety of RNAs such as spliced viral mRNAs, as well as non-coding viral RNAs such as vsiRNAs (viral small interfering RNAs) and asRNA (long anti-sense RNA). The non-coding RNAs together with early proteins Tat, Rev, Nef, and Vpr have regulatory functions of the provirus either inducing or repressing viral transcription. Once the intracellular conditions are favorable to viral proliferation, late proteins like gp41, p24Gag, and other structural viral proteins are produced. In this figure the repressive interaction of this network are represented by pink T-bars, and activating interactions are represented by black arrows. Early and late proteins are shown in light and dark blue, respectively. Components of the host's transcription machinery are shown in purple, yellow, and black.

Current mathematical models of HIV-1 biology have been focused on transmission dynamics, posttreatment control, Vorinostat, and Romidepsin treatments, as well as the relation between reservoir size and reactivation (Hernandez-Vargas, 2017). However, none of these models addressed whether exist other paths to manipulate molecular components of the HIV to enhance latency reversion. Here we used Boolean and ordinary differential equations (ODEs) models to analyze the dynamics of the GRN of provirus to investigate how to reactivate more efficiently viral reservoirs with LRAs. In this network we included the interactions mediated by early viral proteins, vncRNAs, and epigenetic factors that regulate latency in resting CD4+ T-cells (Figure 1). It is important to remark that we used two different mathematical models in order to obtain results that represent the real dynamics of the GRN, independently of the model type chosen. The discrete model was used to calculate global properties of the network (attractors and its basins). The continuous model was used to measure changes in RNAs and protein expression levels of the GRN components. Both models consistently showed that the architecture of the GRN of wild type proviruses favors latency over activation state because of redundant interactions of vncRNAs. Furthermore, the models showed that reactivating effects of LRAs also stimulate the increase of vncRNAs, which reduces proviral protein expression. Finally, the models showed that the use of inhibitors of histone methyltransferases (HMTs) with releasers of P-TEFb, like chaetocin and JQ1 respectively, may increase proviral reactivation even in presence of vncRNAs.

## Materials and methods

**Figure 2:**
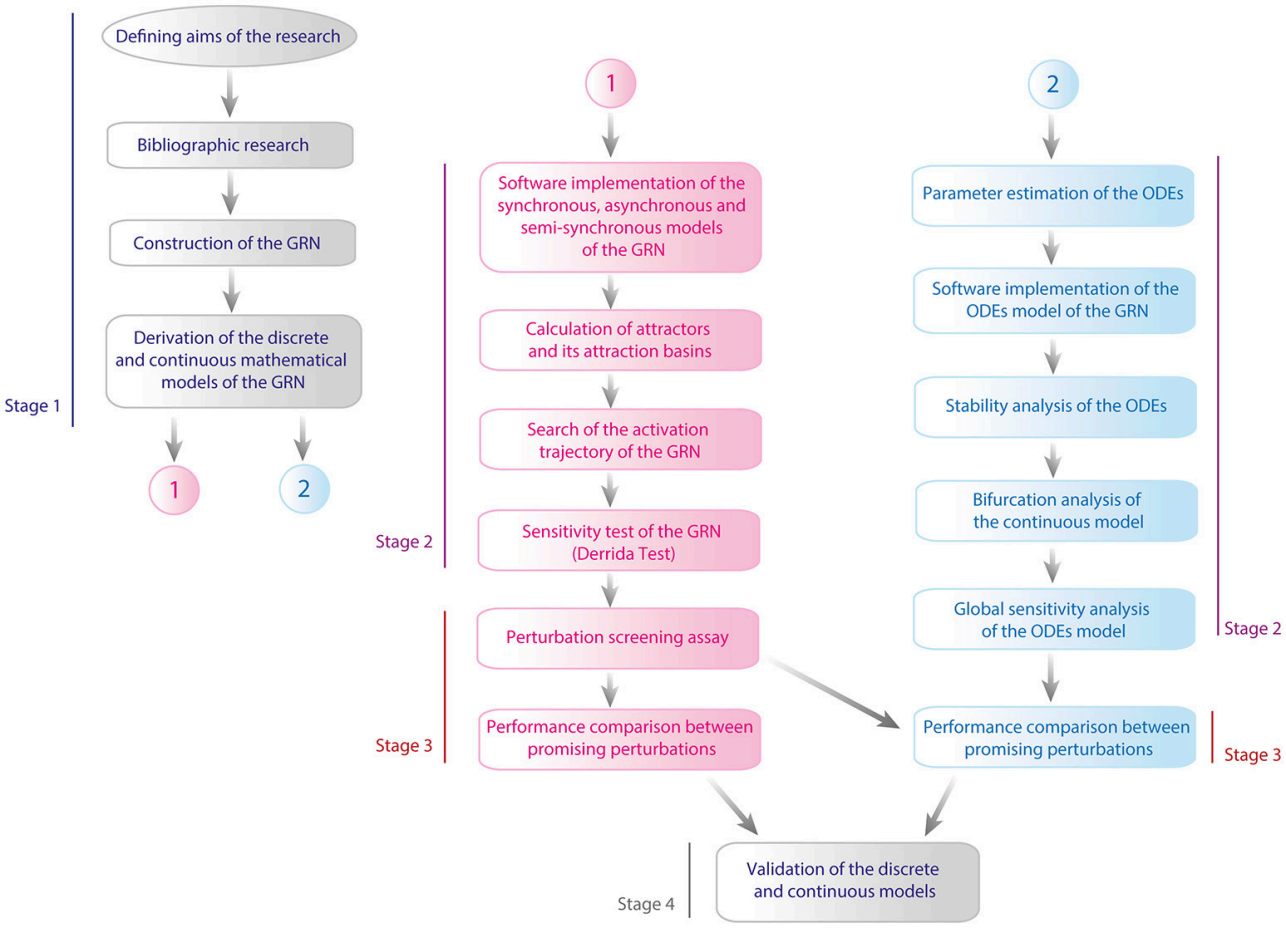
Flux diagram of methodology. This work was executed in four stages which are: (1) Defining the GRN and its models, (2) Mathematical analysis of the models, (3) Perturbation analysis of the models, and (4) Validation. In the figure line blue denoted the first stage, line purple corresponds to stage 2, line red is for stage 3, and finally gray line is assigned to the fourth stage. Pink color is used to represent the methodological steps made with the discrete model and blue color for continuous model.

This work was performed in four stages: (1) Defining the GRN and its models, (2) Mathematical analysis of the GRN models, (3) Perturbation analysis of the models, and (4) Validation. The complete flux diagram of the methodology of this work is shown in Figure 2. During the first stage we constructed the GRN as well as the Boolean and the continuous models, then both models were analyzed separately. For the Boolean model, it was calculated its attractors with their respective attraction basins, then it was calculated the activation trajectory of the GRN and finally, it was evaluated the sensitivity of the model with the Derrida Test. On the other hand, it was calculated the equilibria of the ODEs model and it was evaluated the behavior of trajectories around such points with the analysis of stability, it was then evaluated the effect of particular changes in parameters values with the bifurcation analysis and finally it was evaluated the sensitivity of the model with a global sensitivity analysis. In the third stage it was performed a screening assay to find perturbations that reactivate latent proviruses and it was analyzed the dynamical features of such perturbations with discrete and continuous models. Finally, we validated both models with experimental data available from literature. In the following paragraphs of this section we present details of the protocols used in this work.

### Construction of the network

The GRN was built by compiling information from the literature on the molecular mechanisms that regulate HIV-1 latency inside resting CD4+ T-cells (Figure 1). This GRN included the main interactions of antisense long-non coding RNAs (asRNA), viral small interfering RNAs (vsiRNA), viral small activator RNA (vsaRNA), Tat, Rev, Nef, Vpr, and cellular factors that control gene expression of latent proviruses such as histone deacetylases (HDACs), histone acetyltransferases (HATs), and HMTs.

We incorporated to the GRN the most important molecules and viral components involved in the regulation of provirus gene expression, namely: the concentration of NF-κB, HATs, and HMTs; the activity of viral promoters 5′LTR and 3′LTR; nuclear genomic mRNA of 9 kb, [mRNA9kb(N)]; vsiRNA; vsaRNA; nuclear mRNAs of 4 kb [mRNA4kb(N)] and 2 kb [mRNA2kb(N)]; cytoplasmic genomic mRNA of 9 kb [mRNA9kb(C)], and cytoplasmic mRNAs of 4 kb [mRNA4kb(C)]; and 2 kb [mRNA2kb(C)]; as well as Tat, Rev, Nef, Vpr, asRNA, and the p24 gag protein (p24Gag). Based on the above, we proposed discrete and ODE-based mathematical models to understand the dynamical properties of the GRN. In what follows we present first the discrete model and then the continuous model.

## Discrete model

For the discrete dynamics, the state of the nodes of the network in Figure 1 are represented by a set of binary variables, Σ = {σ_1_, ⋯ , σ_*N*_}, each one taking the value 1 for activation and 0 for inactivation. The value of each variable σ_*n*_ is determined by its *k*_*n*_ regulators, denoted by {σ_*n*_1__, ⋯ , σ_*n*__*k*__*n*___}, through the equation

(1) $$\sigma_{n}\left(t+\Delta t\right)=f_{n}\left(\sigma_{n_{1}}\left(t^{\prime }\right),\sigma_{n_{2}}\left(t^{\prime }\right),\ldots ,\sigma_{n_{k}}\left(t^{\prime }\right)\right),$$

where *f*_*n*_ is a Boolean function that depends on *k*_*n*_ arguments (Table 1). This function is constructed according to the inhibitory or activating nature of the interactions between σ_*n*_ and its regulators (Kauffman, 1969). The discrete time *t* advances in integer steps; the time *t*′ at which the state of the regulators is evaluated is such that *t* ≤ *t* ′ < *t* + Δ*t*, where Δ*t* is the time it takes to σ_*n*_ to respond to a change in its regulators. Traditionally, Equation (1) is implemented simultaneously (synchronously) on all the nodes of the network. In this synchronous case *t*′ = *t* and Δ*t* = 1. In addition to the synchronous update, we also implemented two other updating schemes: asynchronous and semi-synchronous.

**Table 1:** Logic rules that models the GRN.

**Node**	**Logic rule**	
TNFα	=	*input*
*I*_*HMTS*_	=	*input*
*NFκB*	=	(*TNFα*) *OR* (*Tat*) *OR* (*Vpr*) *OR* (*Nef*)
*HMTs*	=	(*I*_*HMTS*_) *OR* (*asRNA*)
*p*′5*LTR*	=	(*NFkB*) *AND NOT* (*HMTs*)
*p*′3*LTR*	=	(*NFkB*) *AND NOT* (*HMTs*)
*RNAs*9*kbN*	=	*p*′5*LTR*
*vsiRNA*	=	(*p*′5*LTR*) *AND NOT* (*Tat*)
*vsaRNA*	=	(*p*′5*LTR*)
*RNAs*4*kbN*	=	*RNAs*9*kbN*
*RNAs*2*kbN*	=	*RNAs*4*kbN*
RNAs2kbC	=	(RNAs2kbN) AND NOT (vsiRNA)
RNAs4kbC	=	(RNAs4kbN) AND (Rev) AND NOT (vsiRNA)
RNAs9kbC	=	(RNAs9kbN) AND (Rev) AND NOT (vsiRNA)
asRNA	=	(p'3LTR) AND NOT (vsaRNA)
Tat	=	(RNAs2kbC) OR (RNAs4kbC)
Rev	=	RNAs2kbC
Nef	=	RNAs2kbC
Vpr	=	(RNAs2kbC) OR (RNAs4kbC)
p24Gag	=	RNAs9kbC

In the asynchronous scheme a permutation with repetition of the network nodes {σ_1_, ⋯ , σ_*N*_} is chosen. Let us denote as *P* = {σ_*p*_1__, σ_*p*_2__⋯σ_*p*_*L*__} this permutation, where *L* ≥ *N*. Then at each time step *t* the nodes of the network are updated one by one following the order of this permutation: first σ_*p*_1__at time $t^{\prime }=t+\frac{1}{L}$, then σ_*p*_2__at time $t^{\prime }=t+\frac{2}{L}$, and so on until σ_*p*_*L*__ is updated at time *t*′ = *t*+1. When σ_*p*_*i*__ is being updated, Equation (1) is applied with $\Delta t=\frac{i}{L}$ and $t^{\prime }=t+\frac{i-1}{L}$. After all the nodes in the permutation have been updated, the time *t* advances one unit and the process is repeated until an attractor is reached.

For the semi-synchronous scheme the set of all network nodes Σ = {σ_1_, ⋯ , σ_*N*_} is partitioned into *S* subsets {*M*_1_, ⋯ , *M*_*s*_} such that ${⋃}_{j=1}^{S}M_{j}=\Sigma$. All the nodes contained in *M*_*j*_ are updated synchronously, but the subsets {*M*_1_, ⋯ , *M*_*s*_} are updated asynchronously: the nodes in *M*_1_ are updated at time $t^{\prime }=t+\frac{1}{S}$, the nodes in *M*_2_ are updated at time $t^{\prime }=t+\frac{2}{S}$, and son on until the nodes in *M*_*S*_ are updated at time *t*′ = *t*+1. When the nodes in *M*_*i*_ are being updated, Equation (1) is applied with $\Delta t=\frac{i}{S}$ and $t^{\prime }=t+\frac{i-1}{S}$. A full time step to go from Δ*t* to *t*+1 consists in the updating of all the subsets {*M*_1_, ⋯ , *M*_*s*_}, one by one in successive order. The construction of the permutation *P* for the asynchronous scheme and the subsets {*M*_1_, ⋯ , *M*_*s*_} for the semi-synchronous one was based on biological phenomenology that reflects the way in which the activation cascade across the network may occur, and it is presented in the Supplementary Material.

It is well-known that the size of the basin of attraction is modified by updating scheme (Gershenson, 2002). The belonging of a network state to a particular basin of attraction strongly depends to updating scheme chosen. This has a biological equivalence, because the cellular environment is noisy and the order of gene expression may occur in different ways. However, there are some network states that always belong to same basin of attraction independently of updating scheme used. We call to this property as *robustness under updating scheme*. We hypothesize that the set of network states with this property are relevant for the biological behavior of the provirus. We call this set of states as *intersection of the network states*. We calculated the intersection of the synchronous, semi-synchronous, and asynchronous to determine the trajectory of activation of the provirus.

### Stability of the boolean model: derrida map test

The discrete model can exhibit two dynamical regimes, ordered and chaotic, and a phase transition between them, the so-called critical point (Aldana, 2003). The characterization of these regimes is given by the behavior of the avalanche of perturbations (produced by stochastic fluctuations, gene knockout, or gene over expression). In the chaotic regime, small perturbations spread throughout the network over time, producing big changes in the network state. Therefore, a network operating in a chaotic regime and submerged in a noisy cellular environment would have very unstable phenotypes. In the order regime, the perturbations die out over time, preventing the network to respond to new changing environmental conditions. In the critical point, the perturbations neither spread to the entire network nor disappear. They typically remain confined within a small fraction of genes. In order to characterize the dynamical regime, we define the normalized Hamming distance *h*(*t*) at time *t* between two network states as:

(2) $$h\left(t\right)=\frac{1}{N}\sum_{n=1}^{N}\left|\sigma_{n}\left(t\right)-{\tilde{\sigma }}_{n}\left(t\right)\right|.$$

In this equation σ_*n*_(*t*) is the state of the *n*th gene at time t in a trajectory starting out from a given initial condition, and ${\tilde{\sigma }}_{n}\left(t\right)$ is the state of the same gene in a different trajectory generated from a different initial condition. The Hamming distance *h*(*t*) can be considered as the normalized size of the avalanche of perturbations generated by differences the two initial conditions. The Derrida map *h*(*t*+1) = *M*(*h*(*t*)) (Derrida and Pomeau, 1986) relates the size of the avalanche at two consecutive time steps. It can be shown that *M*(*h*) is a monotonic increasing function with the property that *M*(0) = 0 (if there is no perturbation at time *t*, there is no perturbation either at time (*t*+1). The slope *S* at the origin of *M*(*h*) is the parameter that characterizes the asymptotic value of the Hamming distance, and hence the network dynamics. *S* is called the average network sensitivity. When *S* < 1 the network is operating in the ordered regime. If *S* > 1, the network exhibits chaotic behavior. If *S* = 1, the network is at the critical point. An intuitive definition (Krawitz and Shmulevich, 2007) is that *S* is the average fraction of genes that change their state at time *t*+1 when a single gene is perturbed at time *t* (Supplementary Material). Therefore, to determine the stability of the network dynamics under perturbations in the initial conditions, one has to compute the network sensitivity *S* from the Derrida map *M*(*h*).

Additionally, one can compute the network stability under *permanent perturbations*. We implemented two types of permanent perturbations: inhibition and overstimulation. For this, we set the state of one node, say σ_*j*_, equal to 0 or 1 all the time (regardless of the state of its regulators). Setting σ_*j*_ = 0 for all time is equivalent to permanently inhibit this node, while setting σ_*j*_ = 1 all the time is equivalent to having this node being constantly expressed. Let us denote as *S*_*j*_ the network sensitivity when σ_*j*_ is permanently perturbed (either inhibited or overstimulated), and as *S*_0_ the sensitivity of the wildtype network. In order to compare the dynamical properties of perturbed proviruses vs. the WT provirus, we define the difference of sensitivity ΔS as:

(3) $$\Delta S=S_{j}-S_{0}.$$

This quantity measures how the network dynamics changes when one of the nodes is permanently perturbed. We performed the same type of analysis for the case in which two nodes σ_*i*_ and σ_*j*_ are simultaneously perturbed in a permanent way, either inhibiting or overstimulating them. This allows us to determine whether between-node epistasis exists that can modify the dynamics of the GRN.

### Probability of viral activation

It is important to note that in the three updating schemes presented here, i.e., synchronous, asynchronous, and semi-synchronous, the network dynamics are deterministic (both the permutation *P* and the subsets {*M*_1_, ⋯ , *M*_*s*_} are fixed). Therefore, in any of these updating schemes, after a transient time the network will fall into an attractor (a periodic pattern of activity). Several attractors may exist, and all the initial conditions that eventually fall into the same attractor are known as the basin of attraction of that attractor. As we show in the Results section, the HIV-1 network has several attractors. In some of them the network dynamics correspond to an active virus (the viral proteins are expressed, particularly p24Gag), whereas in the other attractors the dynamics correspond to an inactive virus (i.e., in the latency state with no expression of p24Gag). We refer to the former as the *active attractors* and to the latter as the *inactive attractors*. In order to determine the probability that a given initial condition leads to the active viral state, we compute the relative size of the activation state (*W*_*on*_) by adding the size of the basins of attraction for all active attractors and dividing this sum by the total number of network states:

(4) $$W_{on}=\frac{1}{\Omega }\sum_{k}\left|B\left(a_{k}\right)\right|,$$

where Ω = 2^N^ is the total amount of network states, and $\left|B\left(a_{k}\right)\right|$ is the size of the basin of attraction of the k–th active attractor. Similarly, the relative size of latency state (Woff) was calculated as follows:

(5) $$W_{off}=1-W_{on}.$$

These metrics determine the frequency of each state of the GRN that leads to an active or inactive attractor.

## Continuous model

In the continuous model, we represent the state of the nodes of the network in Figure 1 by the continuous variables {*x*_1_, ⋯ , *x*_*N*_}, which satisfy the general equation of mass balance (Table 2)

(6) $$\frac{dx_{n}}{dt}=\sum_{k}J_{n_{k}}^{i}-\sum_{j}J_{n_{j}}^{o},$$

where the sums $\underset{k}{\sum }J_{n_{k}}^{i}$ and $\underset{j}{\sum }J_{n_{j}}^{o}$ represent all the fluxes that contribute to increase and decrease *x*_*n*_, respectively. The fluxes are presented in detail in Table 2, and the kinetic parameters (which were obtained from the literature), in the Supplementary Material. The Runge-Kutta 4-5 method was used to solve the system of ODEs.

**Table 2:** Ordinary Differential Equations that models the GRN.

**Node**	**Equation**	**Fluxes**
5′LTR	${\overset{{}^{\circ}}{J}}_{5LTR}=J_{1}-J_{2}$	*J*_1_ = *k*_*b*_(1+*k*_*ac*_[*HATs*]+*k*_*tar*_[*Tat*])([*RNAP*]_*T*_−[3*LTR*]−[5*LTR*])[*NFkB*] *J*_2_ = *k*_*d*_(1+*k*_*me*_[*HMTs*])[5*LTR*]
3′LTR	${\overset{{}^{\circ}}{J}}_{3LTR}=J_{3}-J_{4}$	*J*_3_ = *k*_*b*_(1+*k*_*ac*_[*HATs*])([*RNAP*]_*T*_−[3*LTR*]−[5*LTR*])[*NFkB*] *J*_4_ = *k*_*d*_(1+*k*_*me*_[*HMTs*])[3*LTR*]
RNA9kbN	${\overset{{}^{\circ}}{J}}_{9kbC}=J_{5}-J_{6}-J_{7}-J_{8}$	*J*_5_ = *a*_1_[5*LTR*] *J*_6_ = (*s*_1_+τ+*k*_*RRE*_[*Rev*])[*RNAs*9*kbN*] *J*_7_ = δ_1_[*RNAs*9*kbN*] *J*_8_ = *s*_1_[*RNAs*9*kbN*]
vsiRNA	${\overset{{}^{\circ}}{J}}_{vsiRNA}=J_{9}-J_{10}$	*J*_9_ = *a*_2_[*RNA*_9*kb*_] *J*_10_ = (δ_2_+*r*_1_[*Tat*])[*vsiRNA*]
vsaRNA	${\overset{{}^{\circ}}{J}}_{vsaRNA}=J_{11}-J_{12}$	*J*_11_ = *a*_3_[*RNA*_9*kb*_] *J*_12_ = δ_3_[*vsaRNA*]
asRNA	${\overset{{}^{\circ}}{J}}_{asRNA}=J_{13}-J_{14}$	*J*_13_ = *a*_4_[3*LTR*] *J*_14_ = (δ_4_+*r*_2_[*vsaRNA*])[*asRNA*]
RNA4kbN	${\overset{{}^{\circ}}{J}}_{4kbC}=J_{8}-J_{15}-J_{16}-J_{17}$	*J*_15_ = (τ+*k*_*RRE*_[*Rev*])[*RNAs*4*kbN*] *J*_16_ = δ_1_[*RNAs*4*kbN*] *J*_17_ = *s*_2_[*RNAs*4*kbN*]
RNA2kbN	${\overset{{}^{\circ}}{J}}_{2kbN}=J_{17}-J_{18}-J_{19}$	*J*_18_ = *k*_exp_[*RNAs*2*kbN*] *J*_19_ = δ_6_[*RNAs*2*kbN*]
RNA2kbC	${\overset{{}^{\circ}}{J}}_{2kbC}=J_{18}-J_{20}-J_{21}$	*J*_20_ = δ_7_[*RNAs*2*kbC*] *J*_21_ = *r*_3_[*vsiRNA*][*RNAs*2*kbC*]
RNA4kbC	${\overset{{}^{\circ}}{J}}_{4kbC}=J_{15}-J_{22}-J_{23}$	*J*_22_ = δ_8_[*RNAs*4*kbC*] *J*_23_ = *r*_3_[*vsiRNA*][*RNAs*4*kbC*]
RNA9kbC	${\overset{{}^{\circ}}{J}}_{9kbC}=J_{6}-J_{24}-J_{25}$	*J*_24_ = δ_9_[*RNAs*9*kbC*] *J*_25_ = *r*_3_[*vsiRNA*][*RNAs*9*kbC*]
Tat	${\overset{{}^{\circ}}{J}}_{Tat}=J_{26}+J_{27}-J_{28}$	*J*_26_ = *a*_5_[*RNAs*2*kbC*] *J*_27_ = *a*_6_[*RNAs*4*kbC*] *J*_28_ = δ_10_[*Tat*]
Rev	${\overset{{}^{\circ}}{J}}_{Rev}=J_{29}-J_{30}$	*J*_29_ = *a*_7_[*RNAs*2*kbC*] *J*_30_ = δ_11_[*Rev*]
Nef	${\overset{{}^{\circ}}{J}}_{Nef}=J_{31}-J_{32}$	*J*_31_ = *a*_8_[*RNAs*2*kbC*] *J*_32_ = δ_12_[*Nef*]
Vpr	${\overset{{}^{\circ}}{J}}_{Vpr}=J_{33}+J_{34}-J_{35}$	*J*_33_ = *a*_9_[*RNAs*2*kbC*] *J*_34_ = *a*_10_[*RNAs*4*kbC*] *J*_35_ = δ_14_[*Vpr*]
p24Gag	${\overset{{}^{\circ}}{J}}_{p24Gag}=J_{36}-J_{37}$	*J*_36_ = *a*_11_[*RNAs*9*kbC*] *J*_37_ = δ_15_[*p*24*Gag*]
NF-kB	${\overset{{}^{\circ}}{J}}_{NFkB}=J_{38}-J_{39}$	*J*_38_ = *k*_1_([*NFkB*]_*T*_−[*NFkB*])(*k*_0_[*TNF*]+*k*_2_[*Tat*]+*k*_3_[*Nef*]+*k*_4_[*Vpr*]) *J*_39_ = *k*_−1_[*NFkB*]
HATs	${\overset{{}^{\circ}}{J}}_{HATs}=J_{40}-J_{41}$	*J*_40_ = *k*_5_([*HATs*]_*T*_−[*HATs*])(*I*_*HATs*_+*k*_6_[*Tat*]+*k*_7_[*Vpr*]) *J*_41_ = *k*_−5_[*HATs*]
HMTs	${\overset{{}^{\circ}}{J}}_{HMTs}=J_{42}-J_{43}$	*J*_42_ = *k*_8_([*HMTs*]_*T*_−[*HNTs*])(*I*_*HMTs*_+*k*_9_[*asRNA*]) *J*_43_ = *k*_−8_[*HMTs*]

### Input signals for the GRN

The transcriptional state of provirus can be modified by the NF-κB pathway activated by the *Tumor Necrosis Factor* (TNF) and by chromatin modifications such as acetylation and methylation (Supplementary Material). Those modifications are produced by HMTs and HATs in response to intracellular stimulator signals, represented by I_HMTs_ and I_HATs_, respectively. We take TNF, I_HMTs_, and I_HATs_ as the inputs of the GRN. In the Boolean model these inputs have only two states {0, 1}, which are inactivation and activation respectively. In the ODEs model we use square pulse functions to model the inputs as follows:

For extracellular pulses of TNF:

(7) $$TNF\left(t\right)=\{\begin{array}{c} 1,\;t\in T_{1}\\ 0,\;t\notin T_{1} \end{array}$$

For signals that stimulate HATs activity:

(8) $$I_{HATs}\left(t\right)=\{\begin{array}{c} 1,\;t\in T_{2}\\ 0,\;t\notin T_{2} \end{array}\;$$

For signals that stimulate HMTs activity:

(9) $$I_{HMTs}\left(t\right)=\{\begin{array}{c} 1,\;t\in T_{3}\\ 0,\;t\notin T_{3} \end{array}\;$$

In these equations *T*_1_, *T*_2_, and *T*_3_ are the activation intervals of the input signals (Supplementary Material).

### Stability analysis

The stability analysis of the continuous system was performed using the indirect method of Lyapunov (Supplementary Material). This method starts solving the ODEs in order to find the equilibrium points of the system. Then the ODEs are linearized using the Jacobian matrix to calculate the eigenvalues for all equilibrium points (Supplementary Material). Positive eigenvalues correspond to unstable directions in the phase space, whereas negative eigenvalues correspond to stable directions. If all the eigenvalues corresponding to one equilibrium point are negative, then that point is stable.

### Bifurcation analysis

The bifurcation analysis of the ODEs model was performed by changing one by one the parameters of the model. We focused our attention on the dissociation constants of NF-κB, association and dissociation constants of viral proteins, and degradation constants of RNA's and viral proteins (Supplementary Material). Then, each parameter was varied three orders of magnitude, up and down of their reference value and after that; MATLAB was used to calculate the equilibrium points of the system with their corresponding stability.

### Global sensitivity analysis

The sensitivity of the model against random perturbations was evaluated by assigning a uniform distribution to each parameter in which their reference value was taken as the mean and the standard deviation was assumed to be 10% (Supplementary Material). Then, each distribution was randomly sampled to obtain a set of parameters that were used as the inputs to solve the equations of the model during 1,500 units of time. After 10,000 iterations of this process, the concentration of p24Gag was used as the system's output to analyze the behavior of the model in response to random parameter variation. In all the simulations we set *TNF*(*t*) = 0, *I*_*HATs*_(*t*) = 0 and *I*_*HMTs*_(*t*) = 0.

### Simulating mutants and treatments

The behavior of mutant proviruses during the condensation of viral nucleosomes and T-cells activation was modeled by reducing 10-fold the splicing rate of nuclear mRNA of 4 kb (*s*_1_). The nucleosomal condensation was modeled by providing square pulses of I_HMTs_, and T-cell activation was modeled by increasing the value of the NF-κB activity rate (*k*_1_).

The temporal effects of treatments with histone deacetylase inhibitors (HDACis), PKC agonists, P-TEFb releasers, histone methyltransferase inhibitors (HMTis), and antagonist micro-RNAs (antagomirs) on the GRN dynamics were simulated as follows: to simulate the rise on acetylation due to HDACis, we increased two-fold the reference value of the parameters of HATs activity (*k*_5_). The increase the NF-κB levels due to PKC agonists (Mehla et al., 2010), was modeled by increasing the value of NF-κB levels (*k*_1_) of the ODEs system. Considering that P-TEFb releasers, such as the compound JQ1, enhance the function of Tat to sequester P-TEFb and activate provirus (Li et al., 2013), we modeled this type of LRA by increasing the parameter associated to Tat activity (α_5_). The effects of HMTis and antagomirs were modeled by reducing two-fold the reference value of the parameters of HMTs activity (*k*_8_), synthesis of vsiRNA (α_2_), and asRNA (α_4_).

Mutant proviruses treated with HDACis were simulated by a two-fold increase in the value of the parameter of HATs activity (*k*_5_) as a pharmacological overstimulation and setting to zero the values of the parameters for synthesis of Tat (α_5_ and α_6_), Nef (α_8_), and Vpr (α_9_ and α_10_) as gene knockouts. The inhibition of vncRNAs was simulated by reducing 0-, 2-, 20-, and 200-fold the value of the parameters for the synthesis of vsiRNA (α_2_) and asRNA (α_4_). All parameters cited in this paragraph are listed in Supplementary Material.

Analogously to Equation (3), we define *E*_0_ and *E*_*j*_ as the normalized concentration of p24Gag mRNA for the wildtype network and when σ_*j*_ is perturbed, respectively. The difference

(10) $$\Delta E=E_{j}-E_{0},$$

is a measure of the effect on the viral activation of perturbing the node σ_*j*_ in response to pharmacological treatments.

### Validation of the models

**Figure 3:**
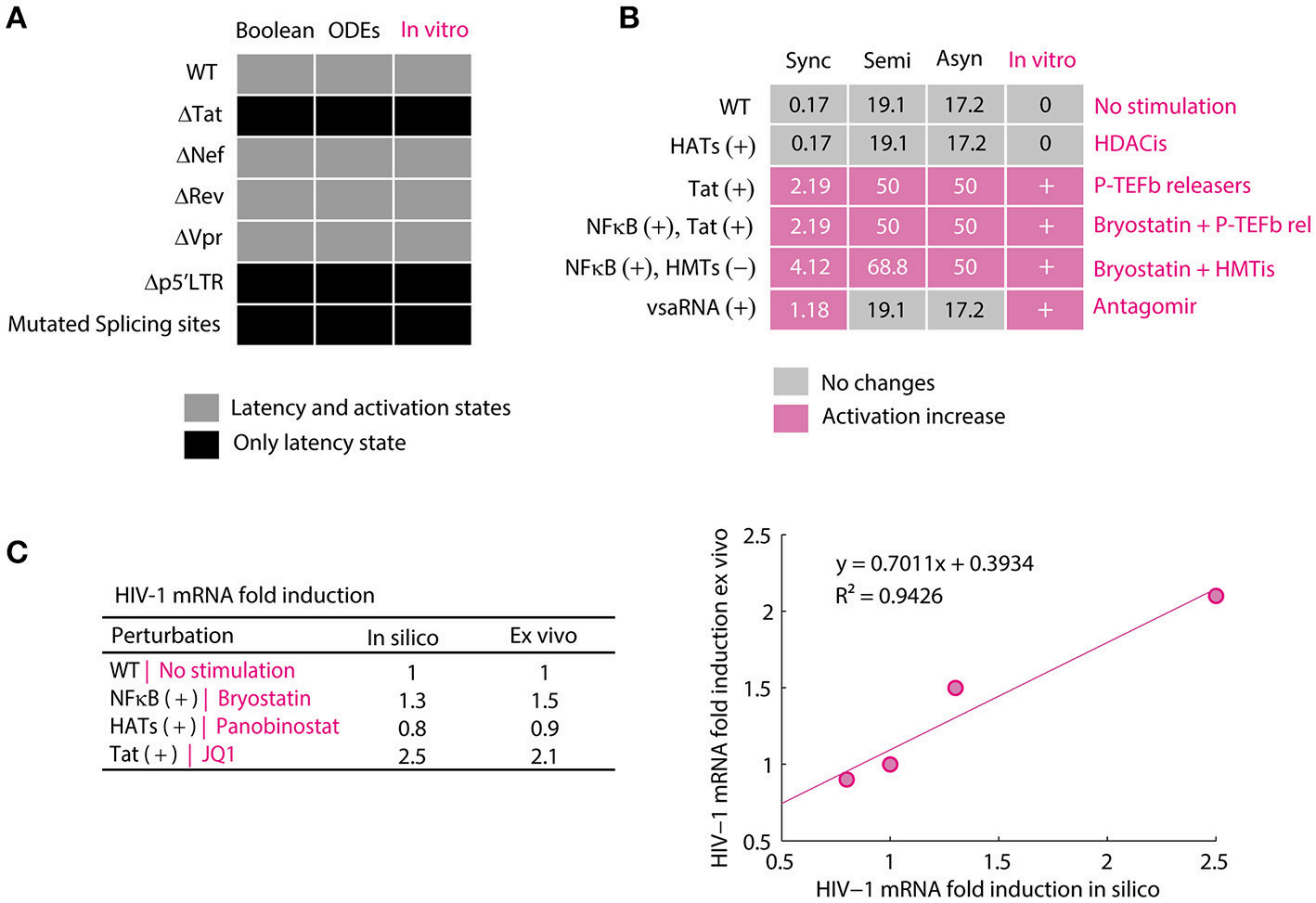
Validation of mathematical models of the HIV-1 GRN. **(A)** Compatibility of the models. In this panel were qualitatively compared the attractors of the Boolean model and the equilibrium points of the ODEs model to provirus behavior observed *in vitro*. The discrete and continuous models present activation and latency states for WT proviruses and deletions of *nef* and *vpr* but only present attractors and equilibrium points for latency state when tat, p5′LTR and splicing sites. This behavior is the same as reported for defective p′5LTR mutants (Ho et al., 2013) and the splicing sites (Purcell and Martin, 1993), deleted *tat* (Verhoef and Berkhout, 1999), *vpr* (Rücker et al., 2004), *rev* and *nef* (Churchill et al., 2007). **(B)** Validation of the Boolean model. In this panel is shown the size of activation state (*W*_*on*_) calculated with the synchronous, semi-synchronous, and asynchronous update scheme. In pink is shown increases of *W*_*on*_ with respect to WT provirus. In the column of *in vitro* observations, “+” represents that there was an increase of viral reactivation because of the treatment and “0” indicates that there were no changes. The data for HDACis was obtained from (Cillo et al., 2014), for P-TEFb releasers from (Li et al., 2013), the use of Antagomirs from (Zhang Y. et al., 2014), combinations of Bryostatin with P-TEFb releasers from (Laird et al., 2015) and combinations of Bryostatin with HMTis from (Bouchat et al., 2012). **(C)** Validation of the ODEs model. In this panel is presented the normalized data of unspliced viral mRNAs levels obtained with the ODEs model and the corresponding values obtained from patients treated with bryostatin (Bullen et al., 2014), panobinostat (Laird et al., 2015), and JQ1 (Laird et al., 2015) vs. their corresponding simulation. Pearson correlation between both data sets showed a positive linear relationship, *p* = 0.0291, *r*_(3)_ = 0.9708, which supports the validity of the model. The standard error of linear regression was 0.1613.

The discrete and continuous models compatibility to reproduce the behavior of HIV-1 GRN was qualitatively evaluated by comparing the dynamical states of each model to the *in vitro* dynamics of provirus genic expression. To perform this, it was calculated the attractors of the discrete model and the equilibrium points of the continuous model for the wild type GRN and mutated networks *p*5′*LTR*(*t*) = 0, *Tat*(*t*) = 0, *Vpr*(*t*) = 0, *Rev*(*t*) = 0, *Nef*(*t*) = 0, and *mRNA*4*kbN*(*t*) = 0. Then, the attractors and the equilibrium points were classified in *activation state* or *latency state* according to their p24Gag expression level (i.e., *latency state* was assigned to attractors and equilibrium points that do not express p24Gag and *activation state* was assigned when p24Gag is expressed). These results were compared to *in vitro* observations reported for the wild type provirus, defective p′5LTR mutants (Ho et al., 2013), deleted *tat* (Verhoef and Berkhout, 1999), *vpr* (Rücker et al., 2004), *rev* and *nef* proviruses (Churchill et al., 2007), as well as deletions on the splicing sites (Purcell and Martin, 1993; Figure 3A). Once compatibility of the models was proved, the discrete model was qualitatively validated by comparing the size of activation state (*W*_*on*_) of the nodes perturbations *HATs*(*t*) = 1, *Tat*(*t*) = 1, *vsaRNA*(*t*) = 1 and the combinations (*NFκB*(*t*) = 1, *HATs*(*t*) = 1), (*NFκB*(*t*) = 1, *HMTs*(*t*) = 0), against their *in vitro* equivalences, which are treatments with HDACis (Cillo et al., 2014), P-TEFb releasers (Li et al., 2013), the use of Antagomirs (Zhang Y. et al., 2014), combinations of Bryostatin with P-TEFb releasers (Laird et al., 2015) and combinations of Bryostatin with HMTis (Bouchat et al., 2012). In Figure 3B is shown the outcome of this comparison, which pointed out that the discrete model is able to predict at qualitative level changes occurred on latency reversion reported *in vitro*. The continuous model was validated by comparing the levels of genomic RNAs obtained *in silico* against *ex vivo* data (Laird et al., 2015). To perform this, it was normalized the levels of p24Gag obtained with the ODEs model for making a linear regression analysis and Pearson correlation with 5% of significance (α = 0.05; Figure 3C). These analysis showed that there is a significant positive relationship between *ex vivo* and *in silico* data sets, *R*^2^ = 0.9426 with *p* < 0.05, which suggest that the ODEs model is able to predict variations over concentration levels of molecular components of the HIV-1 GRN.

## Results

### T-cell activation may not induce expression of the provirus

Razooky and coworkers found evidence suggesting that proviral latency is mainly regulated by the transactivation of 5′LTR mediated by Tat instead of T-cell activation, which implies that latency regulation may be an autonomous process (Razooky et al., 2015). It is in the light of this finding that, the role of epigenetic factors on the performance of Tat's autonomous behavior was investigated. To accomplish this, we analyzed the attractors of the Boolean and its basins of attraction in presence of cellular signals that stimulate epigenetic regulators such as HMTs and HATs, and activators of the NF-κB pathway like TNF. In the three update schemes it was found 12 punctual attractors (the same in the three schemes) which were classified in two groups according to expression of viral proteins as follows: (1) attractors that produce late proteins like p24Gag (*activation attractors*); and (2) attractors that lack protein expression (*latency attractors*; see Table 3).

**Table 3:** Attractors of the HIV Boolean model.

**Nodes**	***a*_1_**	***a*_2_**	***a*_3_**	***a*_4_**	***a*_5_**	***a*_6_**	***a*_7_**	***a*_8_**	***a*_9_**	***a*_10_**	***a*_11_**	***a*_12_**
TNF	0	1	0	1	0	1	0	1	0	1	0	1
IHATs	0	0	1	1	0	0	1	1	0	0	1	1
IHMTs	0	0	0	0	1	1	1	1	0	0	0	0
NF-κB	0	1	0	1	0	1	0	1	1	1	1	1
HATs	0	0	1	1	0	0	1	1	1	1	1	1
HMTs	0	0	0	0	1	1	1	1	0	0	0	0
p5'LTR	0	1	0	1	0	0	0	0	1	1	1	1
p3'LTR	0	1	0	1	0	0	0	0	1	1	1	1
mRNA9kb(N)	0	1	0	1	0	0	0	0	1	1	1	1
vsiRNA	0	1	0	1	0	0	0	0	0	0	0	0
vsaRNA	0	1	0	1	0	0	0	0	1	1	1	1
mRNA4kb(N)	0	1	0	1	0	0	0	0	1	1	1	1
mRNA2kb(N)	0	1	0	1	0	0	0	0	1	1	1	1
mRNA2kb(C)	0	0	0	0	0	0	0	0	1	1	1	1
mRNA4kb(C)	0	0	0	0	0	0	0	0	1	1	1	1
mRNA9kb(C)	0	0	0	0	0	0	0	0	1	1	1	1
asRNA	0	0	0	0	0	0	0	0	0	0	0	0
Tat	0	0	0	0	0	0	0	0	1	1	1	1
Rev	0	0	0	0	0	0	0	0	1	1	1	1
Nef	0	0	0	0	0	0	0	0	1	1	1	1
Vpr	0	0	0	0	0	0	0	0	1	1	1	1
p24Gag	0	0	0	0	0	0	0	0	1	1	1	1
Classification	Latency attractors^*^								Activation attractors			

* *All attractors in which p24Gag was inactive are classified as latency attractors*.

**Figure 4:**
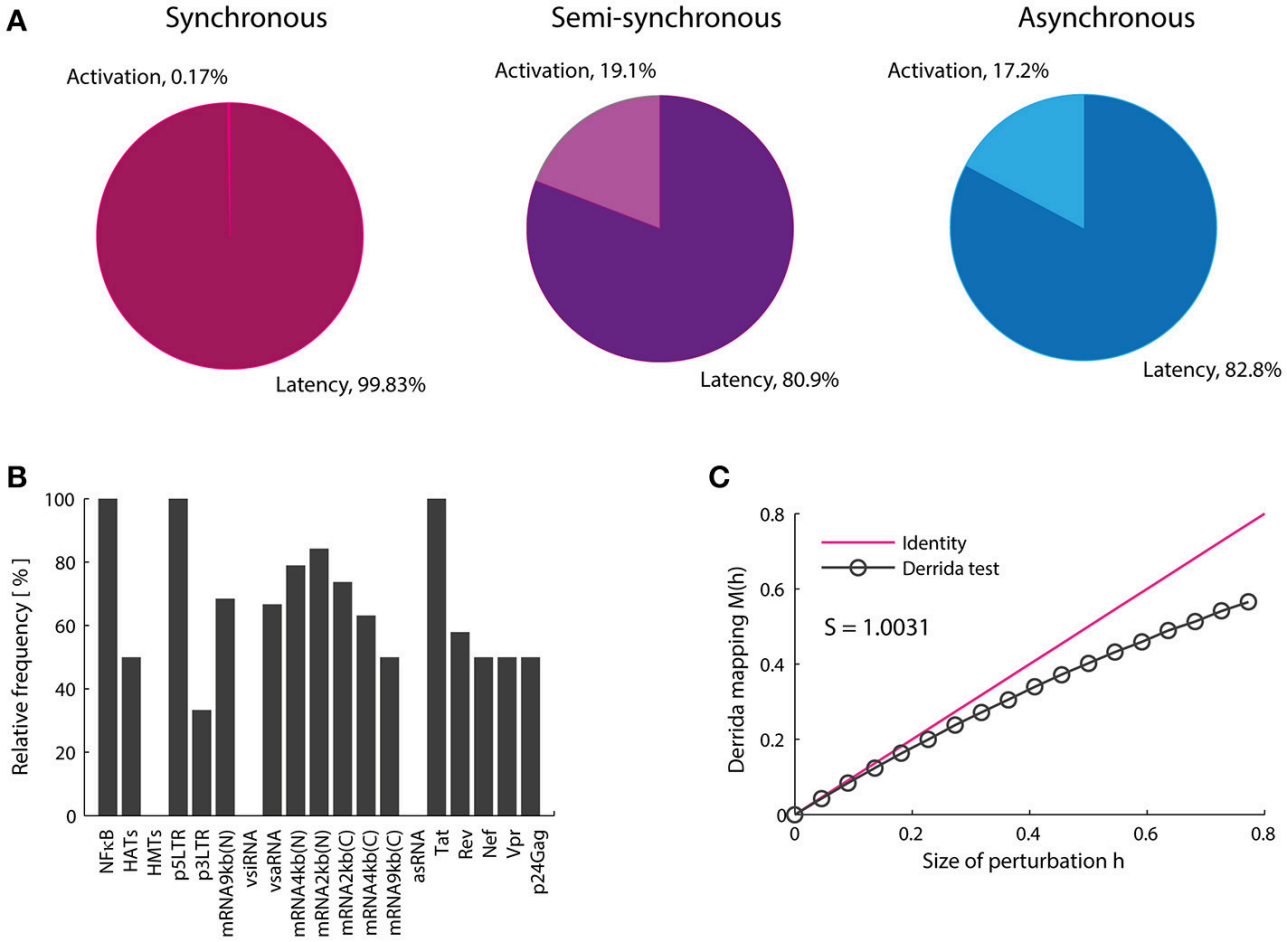
Proviral activation is repressed by vncRNAs and rescued by Tat. **(A)** Relative weight of the activation state (in percentage). In this panel is shown the result of calculating the probability of activation using the synchronous, semi-synchronous, and asynchronous update schemes. In all cases it is shown that latency is favored over activation once provirus is integrated in the host genome. **(B)** Trajectory of activation obtained from intersection of the three update schemes. This panel shows that the presence of NF-kB, p5′LTR, Tat as well as the absence of vncRNAs (i.e., vsiRNA and asRNA) and HMTs are obligatory conditions to activate latent proviruses. **(C)** Sensitivity of the network calculated with Derrida's mapping test. The data obtained from the Boolean model suggested that Tat and vncRNAs are the main proviral regulators of latency and activation.

The Boolean model shows that the activation attractors can be reached with or without cellular stimulation of HATs and TNF (Table 3), which agrees with previous observations that demonstrate the persistence of provirus expression in resting CD4+ T-cells (Razooky et al., 2015). However, this dynamics always requires the absence of the silencing produced by the HMTs activity (Table 3). The probability with which the provirus reaches latency and activation was investigated by calculating the relative size of the activation state (*W*_*on*_) as well as the relative size of the latency state (*W*_*off*_). It was found that *W*_*on*_ is always smaller than *W*_*off*_ (Figure 4A) even when transcription stimulatory signals like HATs and the NF-κB pathway are turned on. These results suggest that even in the context of T-cell activation, provirus may remain latent because of its autonomous dynamics, which is limited by epigenetic silencing.

### Viral non-coding RNAs are essential to regulate latency

Previous reports showed the importance of Tat as the unique virus-encoded regulator of HIV-1 autonomous behavior (Weinberger et al., 2005; Razooky et al., 2015). However, a virus-encoded siRNA that also promotes provirus activation has been found recently (Zhang Y. et al., 2014). Additionally, other virus-encoded regulators, such as vncRNAs that directly repress provirus gene expression have been found (Groen and Morris, 2013; Saayman et al., 2014; Suzuki et al., 2015). Therefore, the role of vncRNAs on the regulation of proviral latency was investigated by searching for common states in all basins of attraction of activator attractors obtained with the three updating schemes (Figure 4B). Using this procedure we found a set of GRN states that abrogate latency (Figure 4B). This set of states indicates a general pattern that results in provirus activation, which agrees with previous reports and requires: high levels of NF-κB (Westendorp et al., 1995), no epigenetic silencing by HMTs (Jordan et al., 2003; du Chéné et al., 2007), genomic integrity of provirus (Ho et al., 2013), high levels of Tat (Weinberger et al., 2005; Razooky et al., 2015), and the absence of repressive vncRNAs (denoted by asRNA and vsiRNA; Figure 4B). This result suggests that Tat and repressive vncRNAs are essential virus-encoded regulators of latency establishment and activation.

### HIV-1 is resistant to drugs and intracellular perturbations

Genetic networks of organisms are able to maintain and adapt their operation in response to environmental changes. Previous studies have shown that the coexistence of robustness and adaptability observed in genetic networks is characteristic of systems operating at the critical point, i.e., at the border of chaos and order (Balleza et al., 2008). This dynamical feature has been reported for genetic networks of *A. thaliana, D. melanogaster, S. cerevisiae, E. coli, B. subtilis* (Balleza et al., 2008) as well as of mice macrophages (Nykter et al., 2008). It has been suggested that criticality is essential to ensure the evolution of any organism (Balleza et al., 2008). We investigated the presence of critical dynamics in the HIV-1 GRN. To do this, the effect of massive perturbations on the GRN was evaluated using the Derrida mapping test. When the network sensitivity *S* for the provirus GRN was computed, it was obtained *S* = 1.0031 which means that the network operates in a critical regime (Figure 4C). Therefore, this network shows equilibrium between robustness and adaptability in resting CD4+ T-cells (Figure 4B). This result suggests that the regulation of the expression of the HIV genome is robust against intracellular perturbations and it can be adapted in response to chronic perturbations, such as those produced during cART or treatments with LRAs. It should be noted that the HIV-1 network has constructed taking into account the activating and inhibitory interactions reported in the literature without considering criticality as a relevant criterion. The result showing that the dynamics of the HIV-1 GRN is so close to criticality is unexpected.

### The architecture of the HIV-1 GRN allows viral rebounds and persistence

**Figure 5:**
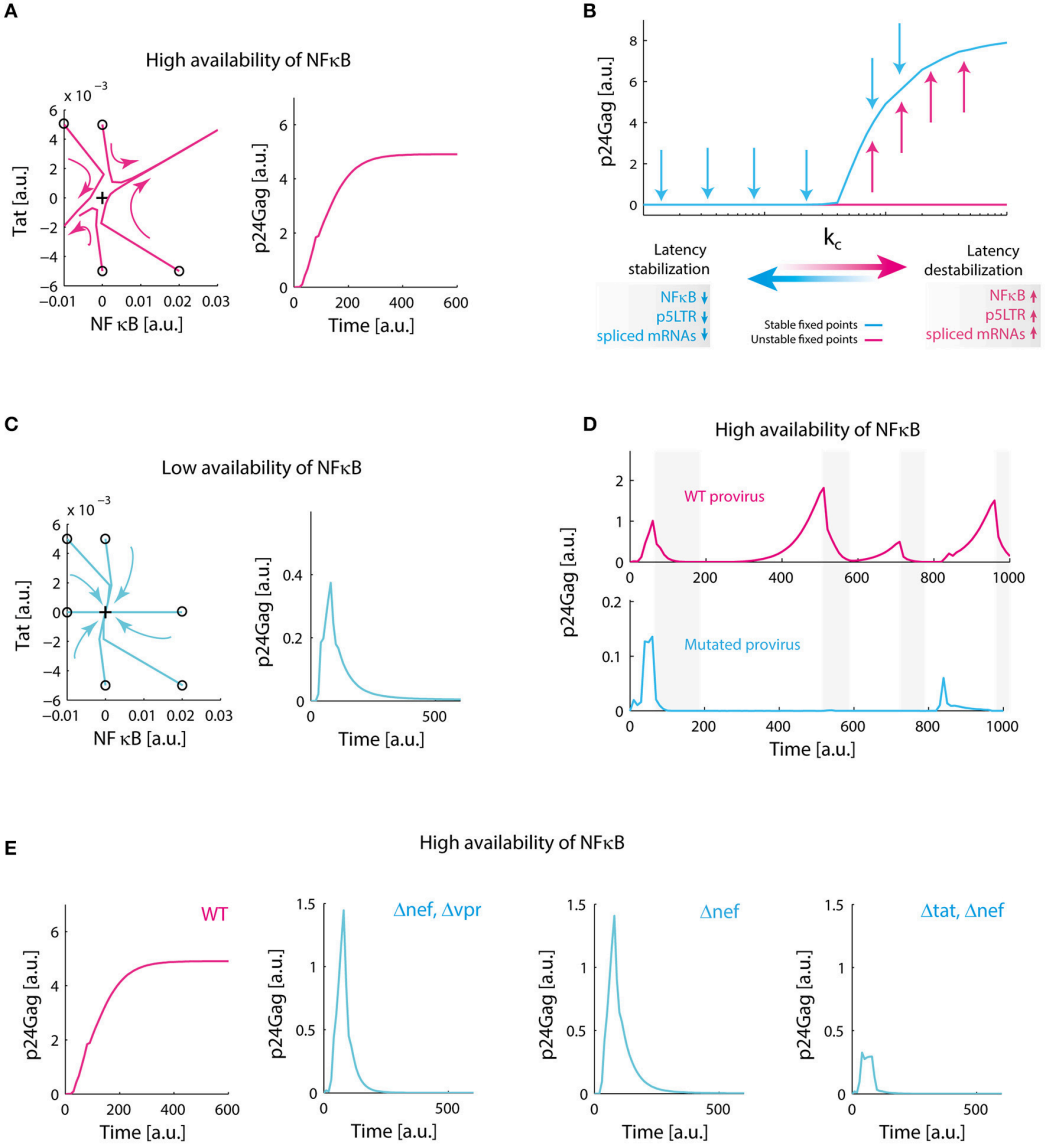
Redundant positive feedback loops of Tat, Nef, and Vpr promote viral persistence. **(A)** Destabilization of latency in the presence of high levels of NF-κB. Phase portrait of the system around the equilibrium point corresponding to latency (black cross), and the temporal performance of the system are shown. In the phase portrait, trajectories of the system are repelled to activation state, on the temporal plot of the system; p24Gag reaches an expression stable state. This simulation was made with our reference value for NF-κB availability (*k*_1_). **(B)** Transcritical bifurcation on the GRN. We found that variations on parameters related to availability of NF-κB, the activity of 5′LTR promoter and the splicing of viral mRNAs change the dynamical behavior of the system. The critical parameters to obtain this bifurcation are included in Supplementary Table 5. **(C)** Stabilization of latency. When we decreased 10-fold NF-κB availability, all trajectories in the phase portrait of the system converge to latency state (black cross), in the temporal plot this can be observed as a transient activation of protein expression that eventually decays. **(D)** Biological role of transcritical bifurcation. In the absence of this bifurcation, defective proviruses decrease their ability to relapse after a period of repression. This simulation was made by decreasing 10-fold the splicing rate of nuclear mRNA of 4 kb (*s*_1_); gray bars indicate nucleosome compaction due to HMTs activity. **(E)** Molecular origin of transcritical bifurcation. Individually, positive feedback loops of Tat, Nef, and Vpr have a transient activity (as observed in panel **C**), however, transcritical bifurcation emerges when loops are combined. Δ*nef* , Δ*vpr*, and Δ*tat* were simulated by setting to zero the synthesis parameters of Tat, Nef, and Vpr (Supplementary Material). Collectively, these data suggest that redundant activation of NF-kB mediated by Tat, Nef, and Vpr ensures proviral reactivation after a period of repression.

Previous observations on the dynamics of Tat's positive feedback loop demonstrated that this circuit is able to amplify transcriptional fluctuations of provirus by itself, and its activity tends to decay toward a latency stable state (Weinberger et al., 2005). It has been proposed that delays on Tat's activity facilitate latency establishment (Weinberger et al., 2005), which could maintain proviral reservoirs during cART (Rouzine et al., 2015). However, it is unknown whether other viral components like Vpr, Nef, and vncRNAs modify the dynamics of Tat's circuit. In this direction, we extended previous findings by analyzing the provirus gene expression dynamics in the presence of Tat and other viral interactions that regulate proviral transcription, such as those mediated by vncRNAs and positive feedback loops of Nef and Vpr (Varin et al., 2003; Liu et al., 2014). To this end, it was used the continuous model to analyze temporal variations of the dynamics of the levels of provirus proteins and RNAs. It was performed the stability analysis of the ODEs model with a set of reference parameters (Supplementary Material), and found two equilibrium points that correspond to *activation* and *latency* states, i.e., the levels of p24Gag were zero for latency state and distinct to zero for activation state (Table 4). In this regard, the stability analysis showed that the activation state was stable and the latency state was unstable (Figure 5A). Then, the sensitivity of the system against fluctuations was evaluated by performing a global sensitivity analysis finding that the dynamics of the system was robust against perturbations (Supplementary Figure 1), and the mean value of p24Gag during activation state was 11.2 with a variance of 7.2. This suggests that once activation state is reached, the provirus expression is resistant to variations of the intracellular environment.

**Table 4:** Equilibrium points of the HIV ODEs model.

**Nodes**	**Latency equilibrium (arbitrary units)**	**Activation equilibrium (arbitrary units)**
NF-κB	0	0.6243
HATs	0	0.2747
HMTs	0	0.1963
p5′LTR	0	0.3379
p3′LTR	0	0.0831
RNA9kb(N)	0	1.0559
vsiRNA	0	0.1056
vsaRNA	0	0.0211
mRNA4kb(N)	0	0.0807
mRNA2kb(N)	0	0.0279
mRNA2kb(C)	0	0.2792
mRNA4kb(C)	0	0.0154
mRNA9kb(C)	0	0.0563
asRNA	0	0.4070
Tat	0	0.1893
Rev	0	0.4035
Nef	0	1.6142
Vpr	0	0.1893
p24Gag	0	5.5836

We searched for parameters that change stability of the equilibrium points of the GRN performing bifurcation analysis. Indeed, it was found a transcritical bifurcation (Figure 5B) on the value of NF-κB activation constant (*k*_1_), parameters related to splicing of viral mRNAs (*s*_1_), and the activity of the 5′LTR promoter (*k*_*b*_). Bifurcation analysis showed that latency is stabilized when the values of these parameters are decreased (Figure 5C). This observation is congruent with *in vitro* reports of conditions that stabilize latency, such as low levels of NF-κB (Westendorp et al., 1995), deficient splicing sites (Purcell and Martin, 1993), and deletions on 5′LTR promoter (Dar et al., 2014) (Table 1). Then, we investigated the possible function of this bifurcation in the context of intracellular infection of HIV-1. To implement this, it was compared the performance of WT provirus vs. mutated provirus that have attenuated splicing rates (10-fold lower of the reference value for *s*_1_) in presence or absence of epigenetic silencing (i.e., when HMTs are active). It was found that transcritical bifurcation allows viral rebounds of the WT provirus after cellular inhibition (Figure 5D), which suggests that persistence may be “hardwired” on the HIV-1 genome. On the other hand, proviruses that lack transcritical bifurcation can be easily controlled by the host's HMTs (Figure 5D). These results suggest that the transcritical bifurcation of the provirus GRN may provide two dynamical behaviors: (1) for repressive transcriptional environments, such as during cART, the provirus latency will be stabilized allowing reservoirs maintenance, and (2) for non-repressive transcriptional environments, the provirus favors a strong activation in order to ensure the production of viral progeny and to counteract the intracellular silencing mechanisms. These properties may explain the viral rebounds after cART and why HIV-1 cannot be silenced by host.

### The activating core of the GRN consists of the positive feedback loops of Tat, Nef, and Vpr

The molecular basis of the transcritical bifurcation was investigated comparing the activity of intact provirus vs. the activity of mutant proviruses. Mutant proviruses were simulated in the continuous model by setting to zero all parameters related to the synthesis of viral proteins Tat, Nef, and Vpr. It was observed that the Tat's positive feedback circuit always produces a stable branch on latency state, which in biological terms is a transient activation followed by latency stabilization dynamics as reported by Weinberger et al. (2005) (Figure 5E). However, combining Tat positive feedback with Vpr and Nef produces the transcritical bifurcation, in which latency can be destabilized (Figure 5B). We also observed that in the absence of Tat the remaining positive feedback loops were able to temporarily perturb latency during stimulation, producing transitory gene activation, but their effect was negligible compared to that observed in the presence of Tat (Figure 5E). Thus, the transcritical bifurcation is sustained by all the positive feedback loops of the viral proteins Tat, Vpr, and Nef (Figure 5E). Considering that all the positive feedback loops of HIV-1 promote NF-κB activation (Figure 1), it is reasonable to think that the redundancy on NF-κB stimulation is the cause of the transcritical bifurcation and its amplifying properties.

### Permanent stabilization of latency occurs more frequently than reactivation

**Figure 6:**
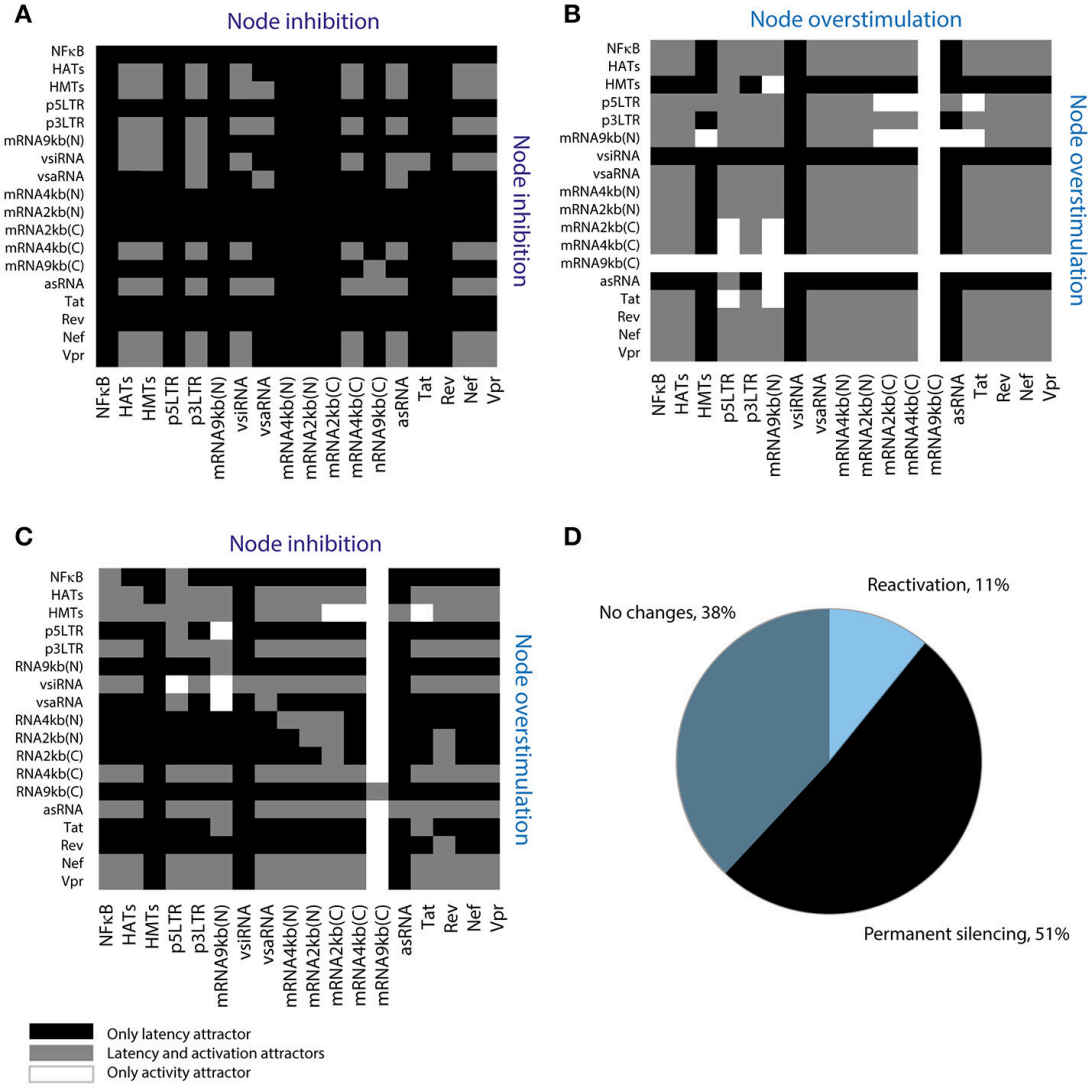
Screening assay for reactivating perturbations. **(A)** Simultaneous inhibition of two nodes of the network. **(B)** Simultaneous overstimulation of two nodes of the network. **(C)** Inhibition and overstimulation of two nodes of the network. **(D)** Summary of screening results. This assay shows that 51% of the perturbations permanently silence provirus' expression; where “reactivation” refers to perturbations that suppress latency attractors, “no changes” refers to perturbations that allow the coexistence of latency and activation attractors, and “permanent silencing” refers to perturbations that abrogate activation attractors.

Recently, it has been proposed that compounds that increase fluctuations of transcriptional basal levels may enhance the performance of LRAs (Dar et al., 2014). Such compounds indirectly target the 5′LTR promoter, increasing its activity. We extended this result by searching for sensitive interactions that could increase proviral reactivation in the presence of LRAs. To this end, it was used the Boolean model to explore all possible perturbations of the provirus GRN by combining inhibition and stimulation of the GRN nodes using a screening assay (Figure 6). It was found that 51% of the perturbations eliminated activation attractors, which suggests that those perturbations are able to induce permanent silencing of the provirus (Figure 6D). On the other hand, it was found that only 28 of the 648 theoretical perturbations can be performed *in vivo* using current LRAs and antagomirs (Table 5). Remarkably, some of these perturbations have not been tested yet. These results suggest that it would be easier to induce the permanent silencing of HIV-1 proviruses rather than reactivating them (Figure 6D).

**Table 5:** Proposed treatments to reverse latency and their current status.

**Perturbation**	**Equivalent treatments**	**References**
HMTs (–)	HMTis	Bouchat et al., 2012
HMTs (–), vsiRNA (-)	HMTis + Antagomirs	^*^
HMTs (–), asRNA (-)	HMTis + Antagomirs	^*^
vsiRNA (–)	Antagomirs	^*^
vsiRNA (–), asRNA (–)	Antagomirs	^*^
asRNA (–)	Antagomirs	Saayman et al., 2014
NF-κB (+)	PKC agonists	Mehla et al., 2010
NF-κB (+), HATs (+)	PKC agonists + HDACis	Laird et al., 2015
NF-κB (+), vsaRNA (+)	PKC agonists + Antagomirs	^*^
NF-κB (+), Tat (+)	PKC agonists + P-TEFb releasers	Laird et al., 2015
HATs (+)	HDACis	Bullen et al., 2014
HATs (+), vsaRNA (+)	HDACis + Antagomirs	^*^
HATs (+), Tat (+)	HDACis+ P-TEFb releasers	Darcis et al., 2015
vsaRNA (+)	vsaRNA	Zhang Y. et al., 2014
vsaRNA (+), Tat (+)	Antagomirs + P-TEFb releasers	^*^
Tat (+)	P-TEFb releasers	Darcis et al., 2015
NF-κB (+), HMTs (–)	PKC agonists + HMTis	Bouchat et al., 2012
NF-κB (+), vsiRNA (–)	PKC agonists + Antagomirs	^*^
NF-κB (+), asRNA (–)	PKC agonists + Antagomirs	^*^
HATs (+), HMTs (–)	HDACis + HMTis	Bouchat et al., 2012
HATs (+), vsiRNA (–)	HDACis + Antagomirs	^*^
HATs (+), asRNA (–)	HDACis + Antagomirs	^*^
vsaRNA (+), HMTs (–)	Antagomirs + HMTis	^*^
vsaRNA (+), vsiRNA (–)	Antagomirs	^*^
vsaRNA (+), asRNA (–)	Antagomirs	^*^
Tat (+), HMTs (–)	P-TEFb releasers + HMTis	^*^
Tat (+), vsiRNA (–)	P-TEFb releasers + Antagomirs	^*^
Tat (+), asRNA (–)	P-TEFb releasers + Antagomirs	^*^

* *Not evaluated yet. The most promising pharmacological perturbations that can be performed to reactivate latent proviruses are included in the table. The corresponding treatment for each perturbation can be implemented as follows: Increasing NF-κB levels [denoted by NF-κB (+)] can be obtained using PKC agonists such as bryostatin (Mehla et al., 2010). Increasing acetylation levels of provirus [denoted by HATs (+)] can be obtained by protecting HATs dependent acetylation with inhibitors of histone deacetylases (HDACis) such as romidepsin or Suberoylanilide Hydroxamic Acid acid (SAHA, Vorinostat) (Reuse et al., 2009; Bullen et al., 2014). Increasing transcriptional effects of Tat [denoted by Tat (+)] can be induced with P-TEFb releasers like JQ1 (Li et al., 2013). Suppression of HMTs activity [denoted by HMTs(–)] can be performed with inhibitors of those enzymes (HMTis) such as chaetocin (Bouchat et al., 2012). Inhibition of vncRNAs, like asRNA and vsiRNA [denoted by asRNA (–) and vsiRNA (–)] can be performed using antagomirs (Yeung et al., 2009; Saayman et al., 2014)*.

### Inhibition of HMTs and stimulation of P-TEFb increases proviral reactivation

**Figure 7:**
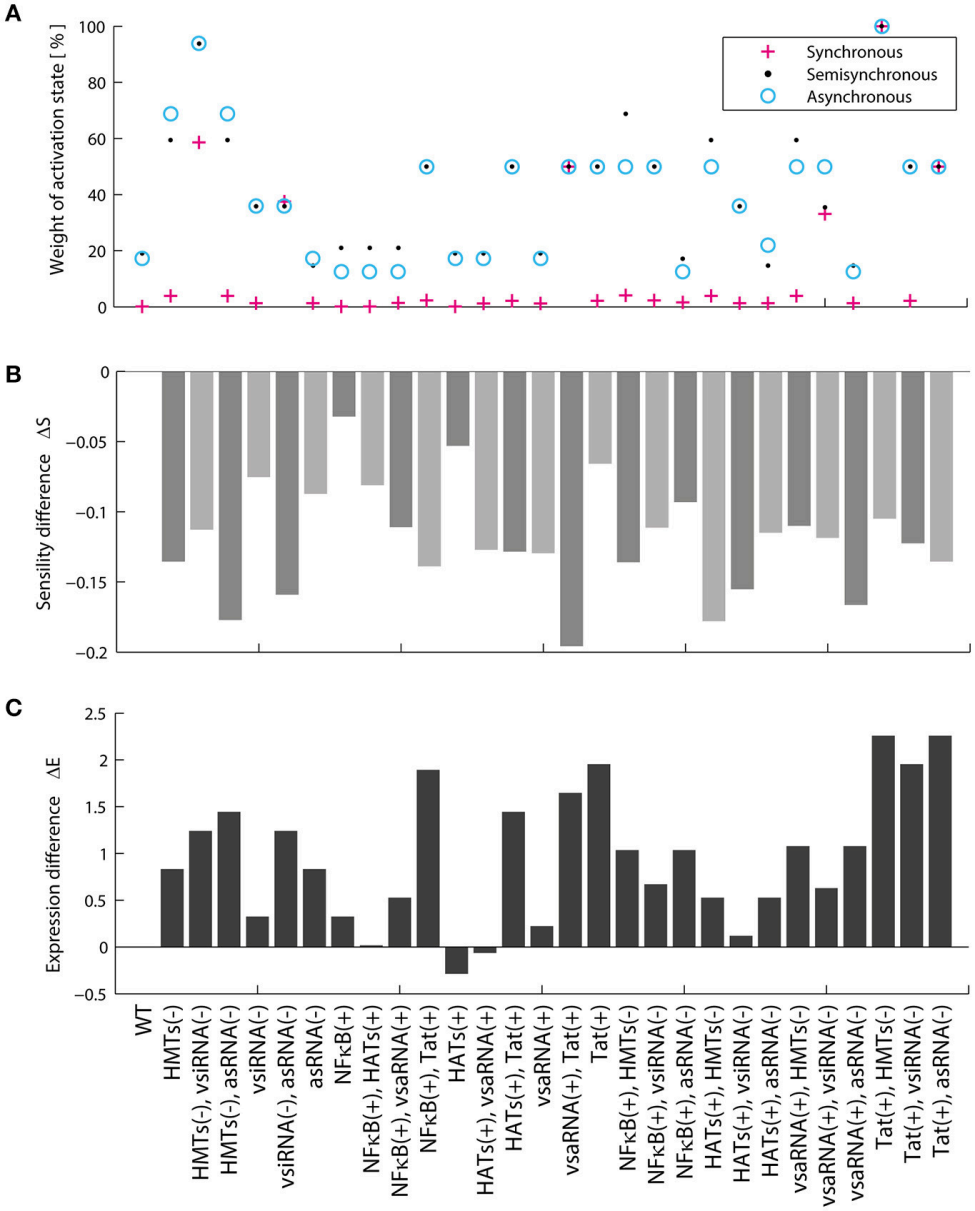
Dynamical features of activating perturbations with LRAs. **(A)** Relative weight of activation state for each activating perturbation. **(B)** Sensitivity difference for each activating perturbation. **(C)** Difference of p24Gag expression for each activating perturbation. In general, all LRAs perturbations increase the weight of the activation state and protein expression.

We then characterized the dynamical properties of 28 promising perturbations produced with LRAs and antagomirs (Table 5). To do this, the dynamical performance of each perturbation was compared to the dynamics of the WT provirus. It was used the Boolean model to calculate the relative size of the activation state (*W*_*on*_) and the difference of sensitivity (ΔS). Similarly, it was used the ODEs model to determine the difference of p24Gag expression (ΔE) for each perturbation. It was found that all reactivation perturbations increased *W*_*on*_, except HATs(+) (Figure 7A) which is equivalent to using HDACis (Table 5). Moreover, all reactivating perturbations decreased network sensitivity (Figure 7B) and the ODEs model showed that all perturbations, except HATs(+), increased the expression of p24Gag (Figure 7C). Remarkably, the discrete model showed that inhibition of HMTs and overstimulation of Tat, i.e., HMTs(–), Tat(+) precludes latency attractors, which means that provirus is always active (Figure 7A). Analogously, the ODEs model showed that HMTs(–), Tat(+) increases ΔE to the maximum (Figure 7C). It is important to note that the pharmacological equivalence of HMTs(–), Tat(+) can be implemented with HMTis and P-TEFb releasers (Li et al., 2013; Table 5). In Table 5 are shown the pharmacological treatment equivalent for the other latency reversing perturbations.

### The performance of LRAs is hindered by vncRNAs

**Figure 8:**
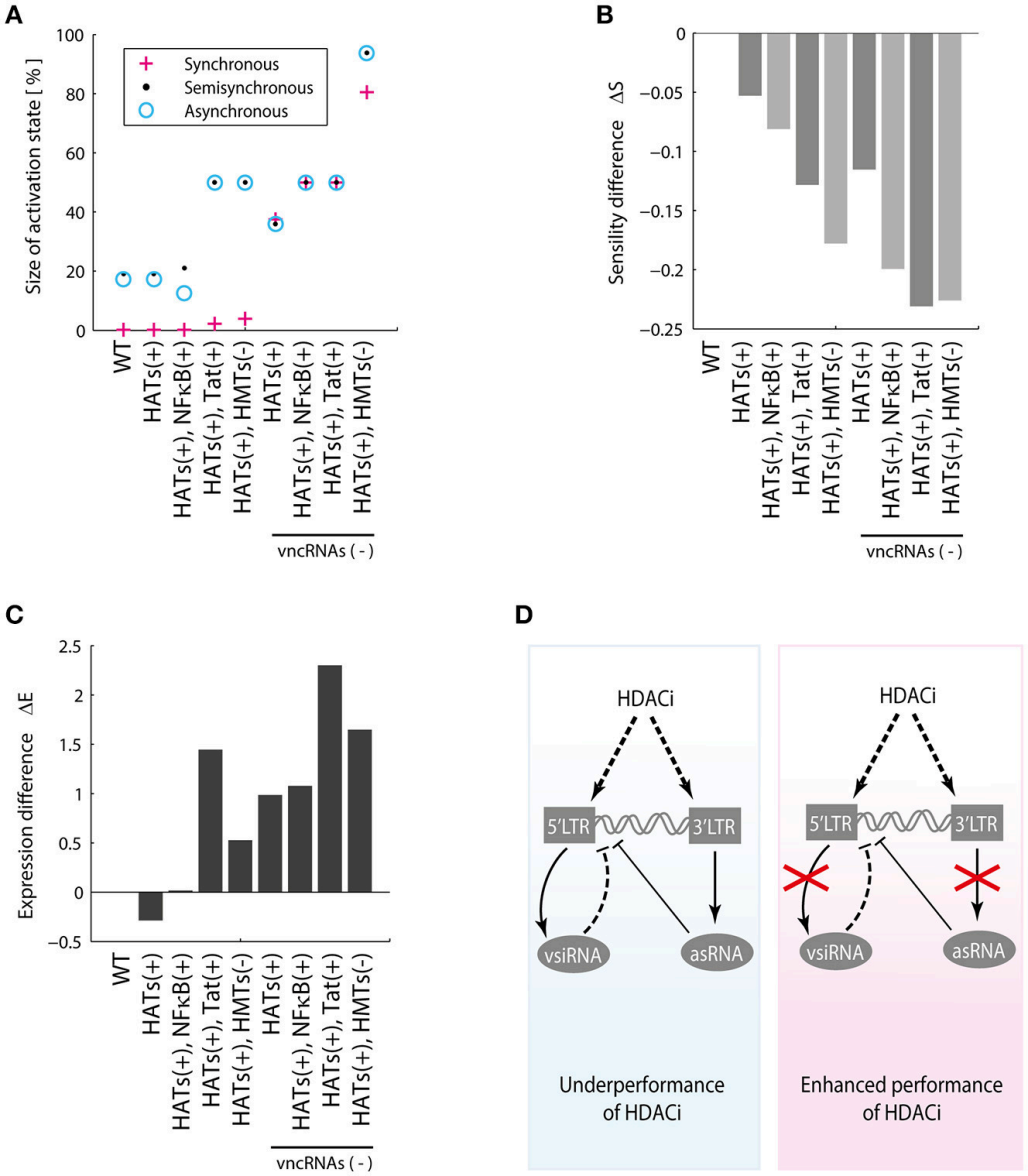
HDACis indirectly increase vncRNAs. **(A)** Relative weight of activation state, **(B)** Sensitivity difference, **(C)** Difference of p24Gag expression with and without vncRNAs, denoted by vncRNAs (–). **(D)** These data suggest that LRAs like HDACis indirectly increase the synthesis of vncRNAs, which hinders their reactivating effects. The suppression of the vncRNAs may enhance the effectiveness of HDACis.

Recent reports showed that HDACis are not effective to reactivate latent proviruses (Bullen et al., 2014; Cillo et al., 2014). In agreement with these reports, the models showed that HDACis do not produce changes in the activation state (Figure 7A) and do not increase p24Gag expression levels (Figure 7C). However, it has been reported that HDACis increase transcription of provirus (Mohammadi et al., 2014). To explain the HDCAis underperformance, the existence of unknown post-transcriptional mechanisms that counteract protein synthesis have been proposed (Mohammadi et al., 2014). Furthermore, it has been reported that HDACis like SAHA (Vorinostat) may increase the levels of cellular non-coding RNAs (Lee et al., 2009). Taken together these observations suggest that HDACis increase provirus transcription as well as the levels of viral and cellular non-coding RNAs, which contributes to silencing protein expression of provirus. We explored this hypothesis by comparing *W*_*on*_, ΔS, and ΔE for each HDACis perturbation with and without vncRNAs (see section Methods). It was found that the suppression of vncRNAs enhances HDACis performance, increasing the values of *W*_*on*_ (Figure 8A), ΔS (Figure 8B), and the expression levels of p24Gag (Figure 8C). These data suggest that HDACis may promote the synthesis of vncRNAs, which may explain why these LRAs increase provirus transcription but not protein expression (Figure 8D).

### Inhibition of vncRNAs is not sufficient to stimulate proviral reactivation

The results just presented indicate that inhibiting vncRNAs could enhance the effect of LRAs (Figure 8D). However, it is not clear whether vncRNAs inhibition can also stimulate the reactivation of mutant proviruses. Therefore, we used the ODEs model to address this question and compared the expression levels of p24Gag in defective provirus treated with HDACis at different intensities of vncRNAs inhibition. It was found that mutant proviruses that lack the Tat protein can be reactivated to a lesser extent than intact proviruses (Supplementary Figure 2). However, defective proviruses that lack two or more positive feedback loops cannot be reactivated, even with the inhibition of vncRNAs (Supplementary Figure 2). These results suggest that inhibition of vncRNAs cannot ensure the total reactivation of proviral reservoirs.

## Discussion and concluding remarks

The long-lived latent reservoirs of HIV-1 are the main barrier to eradicate it. Several efforts to purge viral reservoirs have been performed using LRAs, unfortunately none of them were effective *in vivo* (Bullen et al., 2014). Until now it is not known the causes of the underperformance of LRAs. In this work, we analyzed *in silico* the functioning of the provirus' gene expression in order to investigate the ineffectiveness of LRAs. To this end, we constructed the GRN of provirus and modeled its dynamics using ODEs and logic rules. Both models predicted that vncRNAs are the main negative regulators of the gene expression of provirus and they are also implicated in the underperformance of LRAs. Finally, both models predicted that treatments with HMTis and P-TEFb releasers are the best way to maximize latency reversion.

Traditionally it has been thought that Tat is the only virus-encoded regulator of the HIV latency. However, recent evidence shows that vncRNAs are also essential to control proviral latency. Saayman and colleagues characterized an HIV-encoded long anti-sense RNA which its inhibition triggers reactivation in latently infected cells (Saayman et al., 2014). Zapata and coworkers showed that this long anti-sense RNA is able to silence the gene expression of provirus by stimulating HMTs (Zapata et al., 2017). Thus, we investigated the role of vncRNAs on the dynamics of provirus' gene expression. The first dynamical particularity of the GRN was that the weight of the latency state (*W*_*off*_) was higher than the weight of the activation state (*W*_*on*_), regardless the cell's activation state (Figure 4A). After analyzing the set of intracellular environments that activate the GRN (Figure 4B), we noted that activation requires the presence of Tat and the absence of vncRNAs. Additionally, the inhibition of vncRNAs increased the *W*_*on*_ (Figure 8A). Taken together these results indicate that vncRNAs are the main negative regulators of the provirus' genic expression.

**Figure 9:**
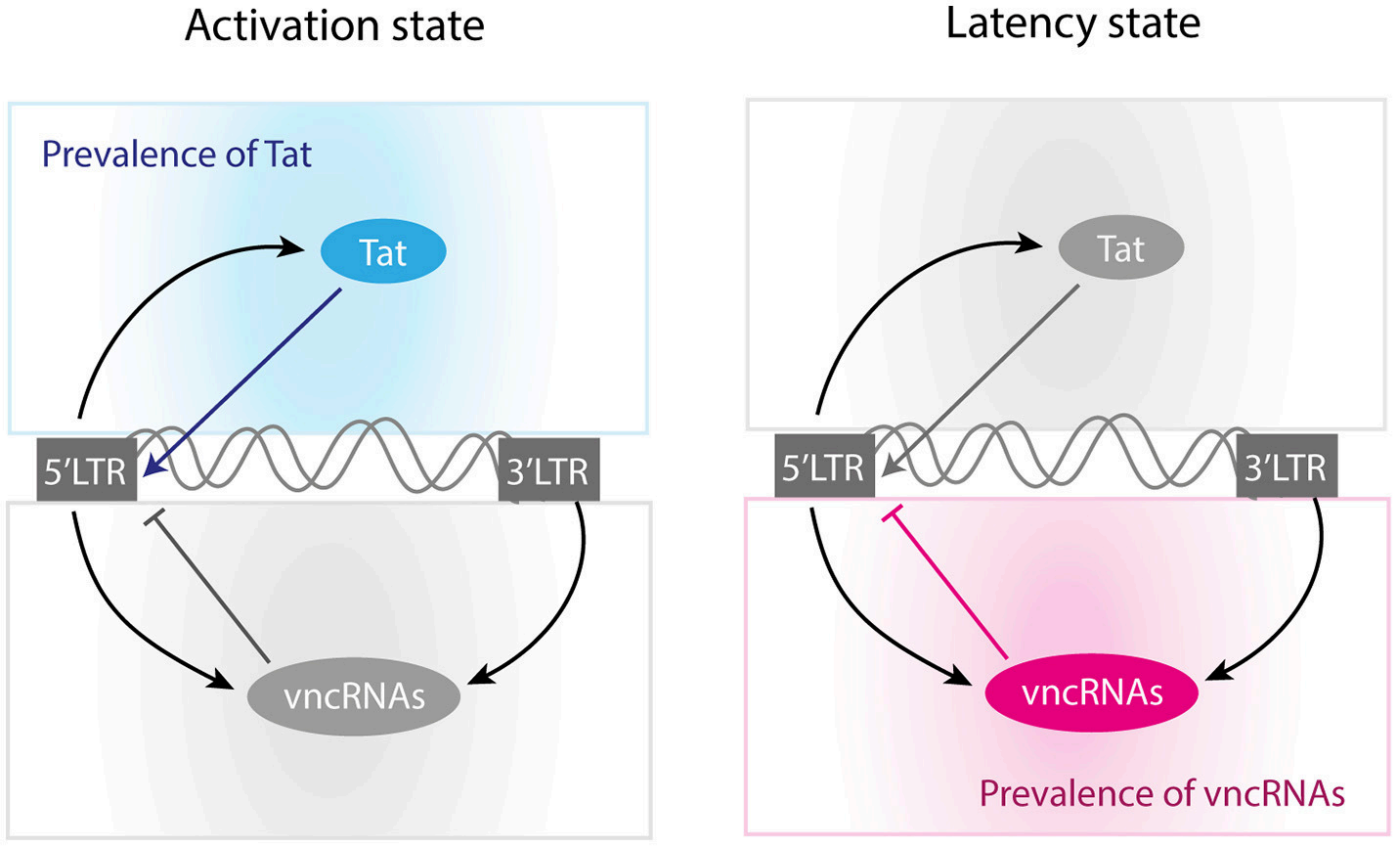
Molecular mechanisms of self-regulation of proviral latency. According to our results, activation state is mainly produced when Tat concentration reaches high levels. On the other hand, the provirus induces its latency when Tat's concentration is not optimal and the levels of vncRNAs are high.

The next question to address was how vncRNAs and Tat operate together to regulate latency. Previous reports demonstrate that Tat's positive feedback loop has a strong transient activation that eventually decays to a stable latency state (Weinberger et al., 2005; Weinberger and Shenk, 2007). The same behavior was observed on the Tat's circuit of the GRN (Figure 5E), as well as in other positive feedback loops mediated by Nef and Vpr (Figure 5E). Interestingly, we found that a transcritical bifurcation appears when these circuits were combined (Figure 5B), and such a bifurcation allows gene expression rebounds after long periods of repression (Figure 5D). It seems likely that the Tat's circuit is enhanced by Nef and Vpr in order to overcome the downregulation of vncRNAs and the host. However, an uncontrolled enhancement of the gene expression of provirus could have negative effects on the viral reservoirs. Rouzine and colleagues found that a high rate of proviral activation avoids the establishment of latent reservoirs, which decreases the prevalence of HIV-1 (Rouzine et al., 2015). They also observed that fluctuations on the transient activity of Tat, decreases the frequency of provirus' activation which stabilizes viral reservoirs (Rouzine et al., 2015). Expanding these observations, our results showed that in addition to Tat's fluctuations, vncRNAs also reduce the activation of provirus. Thus, vncRNAs together with Tat's transient activity may be responsible for the chronic stabilization of latency, condition required to maintain the viral reservoirs (Figure 9).

Furthermore, we investigated the role of vncRNAs on the underperformance of LRAs. The screening assay (Figure 6) showed that 28 perturbations of the GRN can be implemented with LRAs and antagomirs (Table 5), being the combination of HMTis with P-TEFb releasers the most prominent of all. However, perturbations made with HDACis did not increase protein expression of provirus (Figure 7), as reported by Cillo et al. (2014). Mohammadi et al found that HDACis only increase provirus' transcription but did not affect protein expression (Mohammadi et al., 2014). They proposed that this occurs because of post-transcriptional mechanisms that hinder protein expression (Mohammadi et al., 2014). In this direction, our results predicted that the levels of vncRNAs increased in response to HDACis (Figure 8). Hence, it seems likely that treatments with HDACis stimulate proviral transcription as well as vncRNAs, which eventually avoids protein expression. This hypothesis may explain the underperformance of treatments with LRAs reported *in vivo*.

The final question to address was how to enhance the performance of LRAs. The screening assay showed 28 feasible treatments to disrupt latency by using micro-RNAs and current LRAs (Table 5). In this direction the treatment that maximizes the probability to reactivate proviruses (given by the value of *W*_*on*_) uses HMTis and P-TEFb releasers (Figure 7A). The action mechanism of this treatment consists in increasing Tat's levels with P-TEFb releasers while the activity of HMTs is blocked, which is the main downstream target of vncRNAs (Zapata et al., 2017). Therefore, blocking molecular effectors of vncRNAs and enhancing Tat activity is the best way to increase viral reactivation. It is of our interest to test the effectiveness of the treatments proposed in Table 5 with *ex vivo* cultures obtained from HIV patients, in order to determine whether such treatments could be promising for therapeutic implementation.

Nevertheless, our results also showed an interesting scenario that has a distinct approach to control HIV-1. The screening assay showed that 51% of perturbations permanently silence the provirus genic expression (Figure 6D). It is noteworthy to say that the most of perturbations that permanently silence the provirus, inhibit nodes related to proviral transcription such as p5′LTR and unspliced, spliced and partially spliced viral mRNAs (Figure 6A). This implicates that HIV-1 can be permanently controlled by the induction of hypermutation of its genome. A possible mechanism to implement this strategy can be achieved with APOBEC3G, which is the enzyme that naturally hypermutates HIV-1 as a part of intracellular antiviral response. In this context, APOBEC3G is inhibited by Vif in order to allow the progression of HIV-1 infection. However, recent findings suggest that drugs that stimulates ASK1 (apoptosis signal-regulating kinase 1) also restore the APOBEC3G function even in presence of Vif (Miyakawa et al., 2015). Thus, an alternative path to control HIV-1 infection may employ APOBEC3G inducers in conjunction with cART.

Current treatments to reactivate latent proviruses may fail because HIV uses its vncRNAs as negative regulators to maintain latency. Some LRAs like HDACis could increase the levels of vncRNAs, consequently reducing their effectiveness to revert latently infected cells. Our results suggest that the best treatment to avoid the repressive effects of vncRNAs is to use an HMTis like chaetocin, together with P-TEFb enhancers. Treatment that could have potential for efficient reactivation of the HIV-1 provirus should be clinically tested.

## Author contributions

AB and CT-S conceptualized, designed, and performed all computational experiments of this study. RG and JD contributed designing experiments, interpreting, and supervising this study. AB and CT-S wrote the first draft of the manuscript. RG and JD reviewed drafts and approved final version of the manuscript.

### Conflict of interest statement

The authors declare that the research was conducted in the absence of any commercial or financial relationships that could be construed as a potential conflict of interest.

## Abbreviations

Antagomirs: Antagonic Micro-RNAs
ASK1: Apoptosis signal-regulating kinase 1
asRNA: Antisense RNA
cART: Combined Antiretroviral Therapy
GRN: Gene Regulatory Network
HATs: Histone Acetyltransferases
HDACis: Histone Deacetylases Inhibitors
HDACs: Histone Deacetylases
HMTis: Histone Methyltransferases Inhibitors
HMTs: Histone Methyltransferases
LRAs: Latency Reversing Agents
Nef: Negative Effector
NF-κB: Nuclear Factor κB
P-TEFb: Positive Transcription Elongation Factor
Tat: Trans-activator of Transcription
TNF: Tumor Necrosis Factor
Vif: Viral Infectivity Factor
vncRNAs: Viral Non-coding RNAs
Vpr: Viral Protein R
vsaRNA: Viral Small Activator RNA
vsiRNA: Viral Small Interfering RNA.

## References

[B1] Aldana M. (2003). Boolean dynamics of networks with scale-free topology. Phys. D 185, 45–66. 10.1016/S0167-2789(03)00174-X

[B2] Balleza E. Alvarez-Buylla E. R. Chaos A. Kauffman S. Shmulevich I. Aldana M. (2008). Critical dynamics in genetic regulatory networks: examples from four kingdoms. PLoS ONE 3:e2456. 10.1371/journal.pone.0002456 18560561 PMC2423472

[B3] Bouchat S. Gatot J.-S. Kabeya K. Cardona C. Colin L. Herbein G.. (2012). Histone methyltransferase inhibitors induce HIV-1 recovery in resting CD4(+) T cells from HIV-1-infected HAART-treated patients. AIDS 26, 1473–1482. 10.1097/QAD.0b013e32835535f5 22555163

[B4] Bullen C. K. Laird G. M. Durand C. M. Siliciano J. D. Siliciano R. F. (2014). New *ex vivo* approaches distinguish effective and ineffective single agents for reversing HIV-1 latency *in vivo*. Nat. Med. 20, 425–429. 10.1038/nm.3489 24658076 PMC3981911

[B5] Churchill M. J. Chiavaroli L. Wesselingh S. L. Gorry P. R. (2007). Persistence of attenuated HIV-1 Rev alleles in an epidemiologically linked cohort of long-term survivors infected with nef-deleted virus. Retrovirology 4:43. 10.1186/1742-4690-4-43 17601342 PMC1933581

[B6] Cillo A. R. Sobolewski M. D. Bosch R. J. Fyne E. Piatak M. Coffin J. M.. (2014). Quantification of HIV-1 latency reversal in resting CD4+ T Cells from patients on suppressive antiretroviral therapy. Proc. Natl. Acad. Sci. U.S.A. 111, 7078–7083. 10.1073/pnas.1402873111 24706775 PMC4024870

[B7] Cohn L. B. Silva I. T. Oliveira T. Y. Rosales R. A. Parrish E. H. Learn G. H.. (2015). HIV-1 integration landscape during latent and active infection. Cell 160, 420–432. 10.1016/j.cell.2015.01.020 25635456 PMC4371550

[B8] Dar R. D. Hosmane N. N. Arkin M. R. Siliciano R. F. Weinberger L. S. (2014). Screening for noise in gene expression identifies drug synergies. Science 344, 1392–1396. 10.1126/science.1250220 24903562 PMC4122234

[B9] Darcis G. Kula A. Bouchat S. Fujinaga K. Corazza F. Ait-Ammar A.. (2015). An in-depth comparison of latency-reversing agent combinations in various *in vitro* and *ex vivo* HIV-1 latency models identified bryostatin-1+JQ1 and ingenol-B+JQ1 to potently reactivate viral gene expression. PLoS Pathogens 11:e1005063. 10.1371/journal.ppat.1005063 26225566 PMC4520688

[B10] Deeks S. G. (2012). HIV: shock and kill. Nature 487, 439–440. 10.1038/487439a 22836995

[B11] Derrida B. Pomeau Y. (1986). Random networks of automata: a simple annealed approximation. Europhys. Lett. 1, 45–49. 10.1209/0295-5075/1/2/001

[B12] Dinoso J. B. Kim S. Y. Wiegand A. M. Palmer S. E. Gange S. J. Cranmer L. (2009). Treatment intensification does not reduce residual HIV-1 viremia in patients on highly active antiretroviral therapy. Proc. Natl. Acad. Sci. U.S.*A* 106, 9403–9408. 10.1073/pnas.0903107106 PMC2685743 19470482

[B13] du Chéné I. Basyuk E. Lin Y. L. Triboulet R. Knezevich A. Chable-Bessia C.. (2007). Suv39H1 and HP1γ are responsible for chromatin-mediated HIV-1 transcriptional silencing and post-integration latency. EMBO J. 26, 424–435. 10.1038/sj.emboj.7601517 17245432 PMC1783455

[B14] Gershenson C. (2002). Classification of Random Boolean Networks. Computational Complexity; Discrete Mathematics; Dynamical Systems; Cellular Automata and Lattice Gases. Available online at: http://arxiv.org/abs/cs/0208001

[B15] Groen J. N. Morris K. V. (2013). Chromatin, non-coding RNAs, and the expression of HIV. Viruses 5, 1633–1645. 10.3390/v5071633 23812489 PMC3738951

[B16] Hernandez-Vargas E. A. (2017). Modeling kick-kill strategies toward HIV Cure. Front. Immunol. 8:995. 10.3389/fimmu.2017.00995 28894444 PMC5581319

[B17] Hill A. L. Rosenbloom D. I. S. Fu F. Nowak M. A. Siliciano R. F. (2014). Predicting the outcomes of treatment to eradicate the latent reservoir for HIV-1. Proc. Natl. Acad. Sci. U.S.A. 111, 13475–13480. 10.1073/pnas.1406663111 25097264 PMC4169952

[B18] Ho Y. C. Shan L. Hosmane N. N. Wang J. Laskey S. B. Rosenbloom D. I. S.. (2013). Replication-competent noninduced proviruses in the latent reservoir increase barrier to HIV-1 Cure. Cell 155, 540–551. 10.1016/j.cell.2013.09.020 24243014 PMC3896327

[B19] Jordan A. Bisgrove D. Verdin E. (2003). HIV reproducibly establishes a latent infection after acute infection of T cells *in vitro*. EMBO J. 22, 1868–1877. 10.1093/emboj/cdg188 12682019 PMC154479

[B20] Kauffman S. A. (1969). Metabolic stability and epigenesis in randomly constructed genetic nets. J. Theor. Biol. 22, 437–467. 10.1016/0022-5193(69)90015-0 5803332

[B21] Krawitz P. Shmulevich I. (2007). Basin entropy in Boolean network ensembles. Phys. Rev. Lett. 98:158701. 10.1103/PhysRevLett.98.158701 17501391

[B22] Laird G. M. Bullen C. K. Rosenbloom D. I. S. Martin A. R. Hill A. L. Durand C. M.. (2015). *Ex vivo* analysis identifies effective HIV-1 latency–reversing drug combinations. J. Clin. Invest. 125, 1901–1912. 10.1172/JCI80142 25822022 PMC4463209

[B23] Lee E. M. Shin S. Cha H. J. Yoon Y. Bae S. Jung J. H.. (2009). Suberoylanilide Hydroxamic Acid (SAHA) changes microRNA expression profiles in A549 human non-small cell lung cancer cells. Int. J. Mol. Med. 24, 45–50. 10.3892/ijmm_00000204 19513533

[B24] Li Z. Guo J. Wu Y. Zhou Q. (2013). The BET bromodomain inhibitor JQ1 activates HIV latency through antagonizing Brd4 inhibition of Tat-transactivation. Nucleic Acids Res. 41, 277–287. 10.1093/nar/gks976 23087374 PMC3592394

[B25] Liu R. Lin Y. Jia R. Geng Y. Liang C. Tan J.. (2014). HIV-1 Vpr stimulates NF-κB and AP-1 signaling by activating TAK1. Retrovirology 11:45. 10.1186/1742-4690-11-45 24912525 PMC4057933

[B26] Mehla R. Bivalkar-Mehla S. Zhang R. Handy I. Albrecht H. Giri S.. (2010). Bryostatin modulates latent HIV-1 infection via PKC and AMPK signaling but inhibits acute infection in a receptor independent manner. PLoS ONE 5:e11160. 10.1371/journal.pone.0011160 20585398 PMC2886842

[B27] Miyakawa K. Matsunaga S. Kanou K. Matsuzawa A. Morishita R. Kudoh A.. (2015). ASK1 restores the antiviral activity of APOBEC3G by disrupting HIV-1 Vif-mediated counteraction. Nat. Commun. 6:6945. 10.1038/ncomms7945 25901786 PMC4423214

[B28] Mohammadi P. di Iulio J. Muñoz M. Martinez R. Bartha I. Cavassini M.. (2014). Dynamics of HIV latency and reactivation in a primary CD4+ T cell model. PLoS Pathogens 10:e1004156. 10.1371/journal.ppat.1004156 24875931 PMC4038609

[B29] Nykter M. Price N. D. Aldana M. Ramsey S. A. Kauffman S. A. Hood L. E.. (2008). Gene expression dynamics in the macrophage exhibit criticality. Proc. Natl. Acad. Sci. U.S.A. 105, 1897–1900. 10.1073/pnas.0711525105 18250330 PMC2538855

[B30] Purcell D. F. Martin M. A. (1993). Alternative splicing of human immunodeficiency virus type 1 mRNA modulates viral protein expression, replication, and infectivity. J. Virol. 67, 6365–6378. 8411338 10.1128/jvi.67.11.6365-6378.1993 PMC238071

[B31] Razooky B. S. Pai A. Aull K. Rouzine I. M. Weinberger L. S. (2015). A hardwired HIV latency program. Cell 160, 990–1001. 10.1016/j.cell.2015.02.009 25723172 PMC4395878

[B32] Reuse S. Calao M. Kabeya K. Guiguen A. Gatot J. S. Quivy V.. (2009). Synergistic activation of HIV-1 expression by deacetylase inhibitors and prostratin: implications for treatment of latent infection. PLoS ONE 4:e6093. 10.1371/journal.pone.0006093 19564922 PMC2699633

[B33] Romani B. Engelbrecht S. Glashoff R. H. (2010). Functions of Tat: the versatile protein of human immunodeficiency virus type 1. J. Gen. Virol. 91, 1–12. 10.1099/vir.0.016303-0 19812265

[B34] Rouzine I. M. Weinberger A. D. Weinberger L. S. (2015). An evolutionary role for HIV latency in enhancing viral transmission. Cell 160, 1002–1012. 10.1016/j.cell.2015.02.017 25723173 PMC4488136

[B35] Rücker E. Grivel J.-C. Münch J. Kirchhoff F. Margolis L. (2004). Vpr and Vpu are important for efficient human immunodeficiency virus type 1 replication and CD4+ T-cell depletion in human lymphoid tissue *ex vivo*. J. Virol. 78, 12689–12693. 10.1128/JVI.78.22.12689-12693.2004 15507658 PMC525056

[B36] Saayman S. Ackley A. Turner A. W. Famiglietti M. Bosque A. Clemson M.. (2014). An HIV-encoded antisense long noncoding RNA epigenetically regulates viral transcription. Mol. Ther. 22, 1164–1175. 10.1038/mt.2014.29 24576854 PMC4048891

[B37] Siliciano J. D. Kajdas J. Finzi D. Quinn T. C. Chadwick K. Margolick J. B.. (2003). Long-term follow-up studies confirm the stability of the latent Reservoir for HIV-1 in resting CD4+ T cells. Nat. Med. 9, 727–728. 10.1038/nm880 12754504

[B38] Siliciano R. F. Greene W. C. (2011). HIV latency. Cold Spring Harb. Perspect. Med. 1:a007096 10.1101/cshperspect.a007096 22229121 PMC3234450

[B39] Suzuki K. Ahlenstiel C. Marks K. Kelleher A. D. (2015). Promoter targeting RNAs: unexpected contributors to the control of HIV-1 transcription. Mol. Ther. 4:e222. 10.1038/mtna.2014.67 25625613 PMC4345301

[B40] Varin A. Manna S. K. Quivy V. Decrion A. Z. Van Lint C. Herbein G. (2003). Exogenous Nef protein activates NF-κB, AP-1, and c-Jun N-terminal kinase and stimulates hiv transcription in promonocytic cells: role in AIDS pathogenesis. J. Biol. Chem. 278, 2219–2227. 10.1074/jbc.M209622200 12419805

[B41] Verhoef K. Berkhout B. (1999). A second-site mutation that restores replication of a tat-defective human immunodeficiency virus. J. Virol. 73, 2781–2789. 10074125 10.1128/jvi.73.4.2781-2789.1999 PMC104035

[B42] Weinberger L. S. Burnett J. C. Toettcher J. E. Arkin A. P. Schaffer D. V. (2005). Stochastic gene expression in a lentiviral positive-feedback Loop: HIV-1 Tat fluctuations drive phenotypic diversity. Cell 122, 169–182. 10.1016/j.cell.2005.06.006 16051143

[B43] Weinberger L. S. Shenk T. (2007). An HIV feedback resistor: auto-regulatory circuit deactivator and noise buffer. PLoS Biol. 5:e9. 10.1371/journal.pbio.0050009 17194214 PMC1717016

[B44] Westendorp M. O. Shatrov V. A. Schulze-Osthoff K. Frank R. Kraft M. Los M.. (1995). HIV-1 Tat potentiates TNF-induced NF-Kappa B activation and cytotoxicity by altering the cellular redox state. EMBO J. 14, 546–554. 7859743 10.1002/j.1460-2075.1995.tb07030.x PMC398112

[B45] Yeung M. L. Bennasser Y. Watashi K. Le S. Y. Houzet L. Jeang K. T. (2009). Pyrosequencing of small non-coding RNAs in HIV-1 infected cells: evidence for the processing of a viral-cellular double-stranded RNA hybrid. Nucleic Acids Res. 37, 6575–6586. 10.1093/nar/gkp707 19729508 PMC2770672

[B46] Zapata J. C. Campilongo F. Barclay R. A. Demarino C. (2017). The human immunodeficiency virus 1 ASP RNA promotes viral latency by recruiting the polycomb repressor complex 2 and promoting nucleosome assembly. Virology 506, 34–44. 10.1016/j.virol.2017.03.002 28340355 PMC5505171

[B47] Zhang Q. Bhattacharya S. Conolly R. B. Clewell H. J. Kaminski N. E. Andersen M. E. (2014). Molecular signaling network motifs provide a mechanistic basis for cellular threshold responses. Environ. Health Perspect. 122, 1261–1270. 10.1289/ehp.1408244 25117432 PMC4256703

[B48] Zhang Y. Fan M. Geng G. Liu B. Huang Z. Luo H.. (2014). A novel HIV-1-encoded microRNA enhances its viral replication by targeting the TATA box region. Retrovirology 11:23. 10.1186/1742-4690-11-23 24620741 PMC4007588

