# Supplementary material for: Dynamics of the Gene Regulatory Network of HIV-1 and the Role of Viral Non-coding RNAs on Latency Reversion
Source: Front Physiol. 2018 Sep 28;9:1364. doi: 10.3389/fphys.2018.01364 (PMC6172855; doi:10.3389/fphys.2018.01364)
Supplement: Supplementary file 1 [file Table_1.DOCX]

Supplementary Material

Dynamics of the gene regulatory network of HIV-1 and the role of viral non-coding RNAs on latency reversion

Antonio Bensussen^*‡^, Christian Torres-Sosa^‡^, Ramón A. González, José Díaz

*** Correspondence:** bensussenantonio@gmail.com

^‡^These authors contributed equally to this work

| **Contents** |  |  |
| --- | --- | --- |
| Details of the gene regulatory network of HIV-1 provirus |  | 5 |
| Derivation of the Boolean model |  | 6 |
| Details of the Boolean model analysis |  | 8 |
| Derivation of the ODEs model |  | 14 |
| Details of the ODEs model analysis |  | 23 |
| Supplementary References |  | 26 |
|  |  |  |
| **List of Figures** |  |  |
| Supplementary Figure 1. Outcome of the global sensitivity analysis of the ODEs model |  | 2 |
| Supplementary Figure 2. Effect of vncRNAs suppression in defective proviruses |  | 3 |
| Supplementary Figure 3. Difference between the three update scheme |  | 4 |
| **List of Tables** |  |  |
| Table A. Update sequence of the semi-synchronous scheme |  | 9 |
| Table B. Update sequence of the asynchronous scheme |  | 10 |
| Table C. Parameters of the ODEs model and their source |  | 21 |
| Table D. Stability of equilibrium points of the HIV ODEs model |  | 25 |


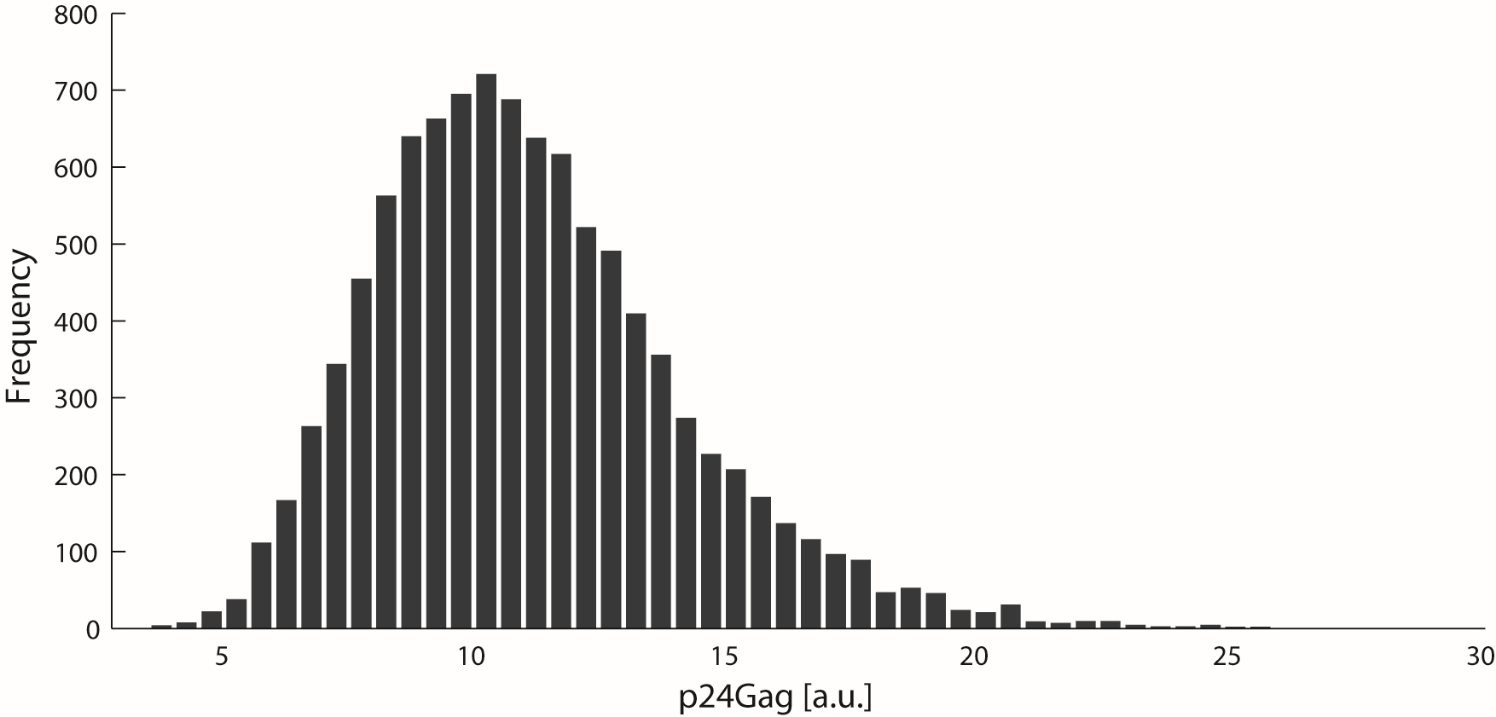


**Supplementary Figure 1**. Outcome of the global sensitivity analysis of the ODEs model. The histogram was made with the output values (p24Gag) of 10,000 simulations that use different samples of the model parameter values in a time interval equivalent to 1,500 arbitrary units of time. In response to the variation of parameters, the output values of the system are well bounded. In addition, it is infrequent that parameter variation triggers the maximum values of the output, which indicate that the system is not sensitive to parameter fluctuations.


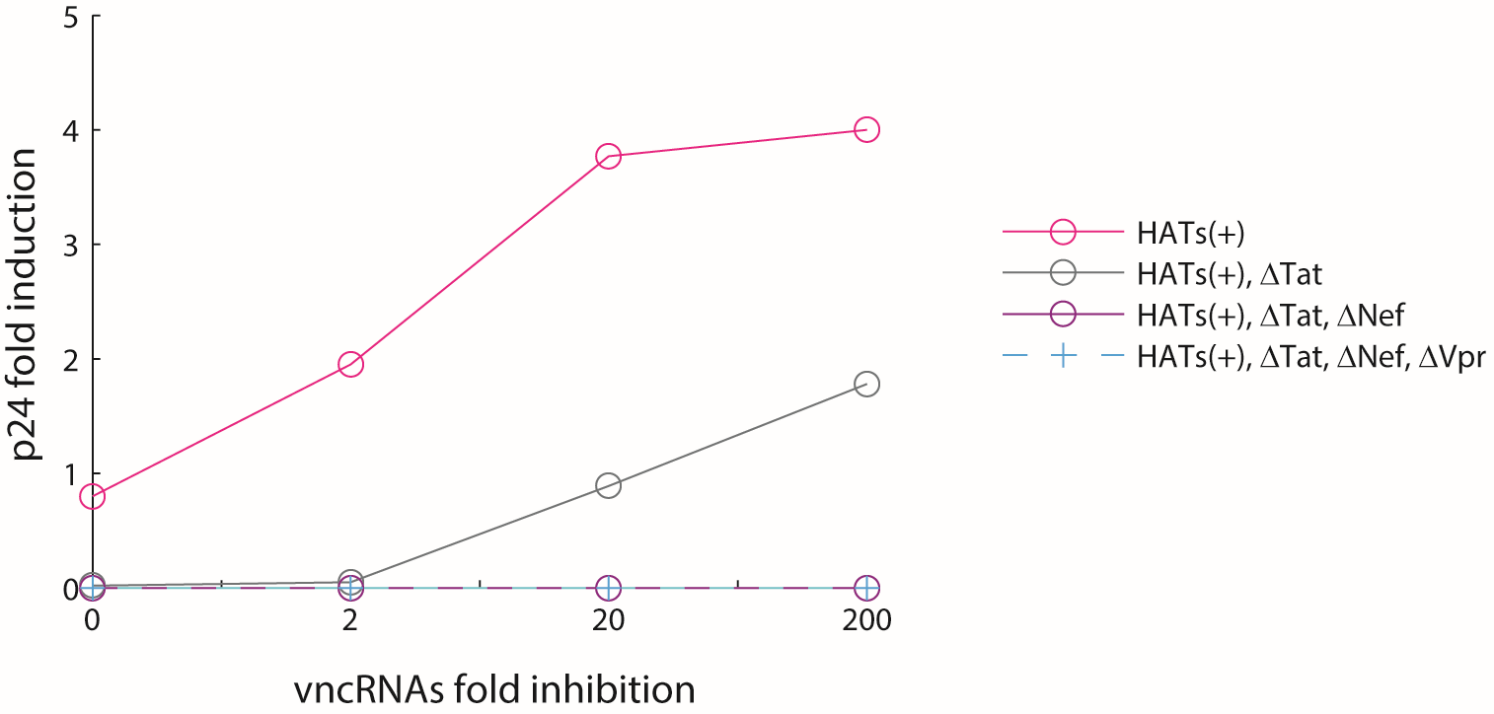


**Supplementary Figure 2**. Effect of vncRNAs suppression in defective proviruses. We simulated the effect of suppressing vncRNAs during administration of HDACis in defective proviruses. The simulations show that proviruses that lack Tat and Nef cannot be reactivated.


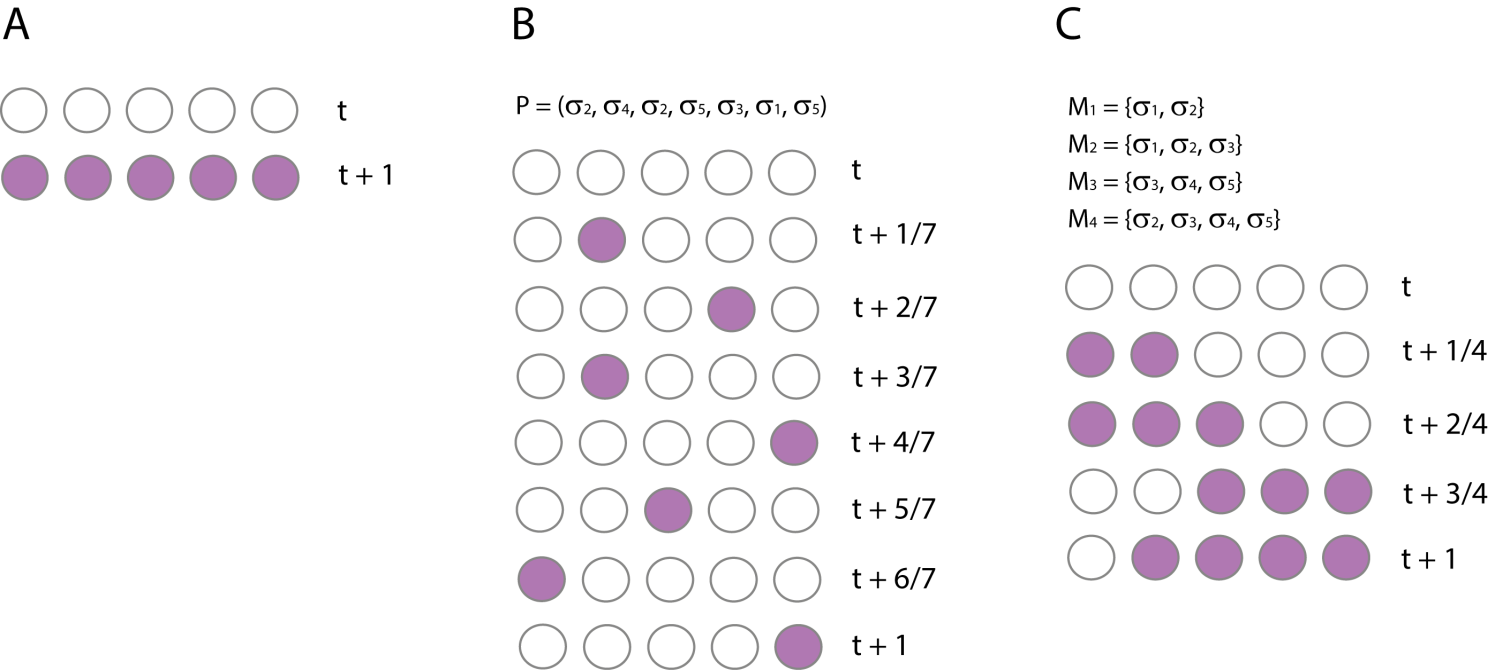


**Supplementary Figure 3.** Difference between the three update scheme. In this figure, each ball represent a node. At the time $t$, all nodes remain in a particular state (1 or 0). This is shown with white balls. When a node is selected to update its respective state is painted with violet color. **A)** In the synchronous update, all the genes are selected for updating at the time $t+1$. **B)** This is asynchronous update. The set $P$, represent the nodes that will be selected for update in different times. In this case, some nodes will be selected more than once. **C)** This is semi-synchronous update. Firstly, we define sets of nodes, say $M_{1},\ldots,M_{4}$. All the nodes that belong to the same set, are updated under synchronous scheme at the same time.

Details of the gene regulatory network of HIV-1 provirus

After integration of the HIV on the host genome, its expression is regulated by chromatin remodeling factors such as HATs, HMTs and HDACs. These enzymes modulate the exposure of viral promoter 5’LTR to transcriptional factors like Sp1, NF-AT and NF-κB, either by relaxation or condensation of the viral nucleosomes (1). In absence of immune stimulation, the 5’LTR promoter produces genomic RNAs of 9 kb (RNA9kb) and truncated RNAs (2,3). After their synthesis, genomic RNAs generate partially spliced RNAs of 4 kb (RNA4kb) and fully spliced RNAs of 2 kb (RNA2kb) (2,3). Simultaneously, truncated and intact viral RNAs are processed to generate a viral small activating RNA (vsaRNA) (4) as well as viral small interfering RNAs (vsiRNA) (5,6) that regulate the gene expression of provirus.

Fully spliced RNAs of 2kb are then exported to the cytoplasm, where they are translated into the viral proteins Tat, Rev, Nef and Vpr (3,7). Tat transactivates the 5’LTR promoter by recruiting P-TEFb and avoids repression mediated by vsiRNA (6,8); Rev facilitates export of the genomic and partially spliced viral RNAs to the cytoplasm (9) and Nef modifies the intracellular environment to protect the cell against the immune system (10). Tat (11) and Vpr (12) interact with HATs to make more accessible the 5’LTR to transcription factors. Tat, Vpr and Nef activate the NF-κB pathway (13–15). On the other hand, it was reported that the *nef*-3’LTR region has an anti-sense promoter that controls the expression of a viral anti-sense long non-coding RNA (16) (asRNA). This asRNA recruits HMTs to the 5’LTR promoter in order to block viral transcription (16).

It was observed that small RNAs that target promoter regions activate gene expression by interacting with cellular anti-sense long non-coding RNAs (lncRNAs) (17,18). This interaction allows lncRNAs to direct chromatin remodeling factors to the targeted promoter in order to activate gene expression (17,18). We noted that HIV-1 encodes two components of this cellular mechanism, which are a small activating RNA that targets 5’LTR promoter (4) and asRNA that directs chromatin remodeling factors to the 5’LTR (16). Based on this observation we propose that this mechanism of cellular gene regulation can also be applied for the virus.

When the equilibrium between activator and repressor interactions is broken the provirus initiates replication. Partially spliced and genomic RNAs are exported to the cytoplasm by Rev, to express other regulatory proteins such as Vpr, Tat, Vpu, Vif, as well as precursor proteins like Gag-Pol, Gag and Env (3). Finally, the cleavage of such precursors produces other accessory proteins like p24Gag, reverse transcriptase (RT), integrase (INT), glycoprotein 120 (gp120) among others (19,20), allowing the assembly of new viral particles. Figure 1 shows the HIV-1 gene expression network constructed from these data.

Derivation of the Boolean model

We transformed the biological information of the network into logic rules. The general procedure that we used consists in two steps: 1) determine the inputs of one node and 2) establishing conditions in which one node will be activated by its inputs.

Based on this general procedure, NF-κB has four inputs which are: TNFα, Tat, Vpr and Nef. NF-κB can be activated by TNFα alone (21) or in conjunction with Tat, Nef or Vpr. The same is true for Tat (13), Nef (14) and Vpr (15). Then, the logic rule for NF-κB is:

$NF-\kappa B= \left( TNF\alpha\right) OR \left( Tat \right) OR \left( Vpr \right) OR (Nef)$ [S1]

The HMTs have two inputs: cellular inducers of HMTs (I_HMTs_) and asRNA. I_HMTs_ is a representation of all intracellular events that may activate HMTs, this input can activate HMTs with or without asRNA (16). Then, the logic rule for HMTs is:

$HMTs=\left( I_{HMTS} \right) OR \left( asRNA \right)$ [S2]

The activity of 5’LTR promoter depends on HMTs, NF-κB and Tat. This promoter is fully active when NF-κB and Tat (22) are present and there is no repression by HMTs (23), thus:

$p'5LTR=(NFkB) AND NOT (HMTs)$ [S3]

On the other hand, the activity of 3’LTR promoter depends on HATs, HMTs and NF-κB. This promoter requires the absence of HMTs repression and the presence of NF-κB (16), therefore:

$p'3LTR=(NFkB) AND NOT (HMTs)$ [S4]

The synthesis of nuclear mRNAs of 9 kb only depends on the activity of 5’LTR promoter (3):

$RNAs9kbN=p'5LTR$ [S5]

The synthesis of vsiRNA depends on the activity of the 5’LTR (24) and the absence of Tat (6), then:

$vsiRNA=\left( p'5LTR \right) AND NOT (Tat)$ [S6]

The synthesis of vsaRNA depends on the activity of the 5’LTR promoter (4), then:

$vsaRNA=(p'5LTR)$ [S7]

The splicing of mRNA 9kb to form spliced mRNAs only depends of nuclear mRNA 9kb (3), then:

$RNAs4kbN=RNAs9kbN$ [S8]

On the other hand, the formation of full spliced mRNAs of 2 kb depends on mRNA of 4 kb (3), thus:

$$RNAs2kbN=RNAs4kbN$$

[S9]

Cytoplasmic functions of mRNAs of 2 kb depends of its transport from the nucleus to de cytoplasm (3) and the absence of Tat (6):

$RNAs2kbC= (RNAs2kbN) AND NOT (vsiRNA)$ [S10]

Cytoplasmic functions of mRNAs of 4 kb depend of its transport to the cytoplasm by Rev (9) as well as the absence of vsiRNA, thus:

$RNAs4kbC= (RNAs4kbN) AND (Rev) AND NOT (vsiRNA)$ [S11]

Similarly to full genomic mRNA:

$RNAs9kbC= (RNAs9kbN) AND (Rev) AND NOT (vsiRNA)$ [S12]

The synthesis of asRNA occurs when promoter 3’LTR (16) is active and there is not vsaRNA (4,18):

$asRNA= (p'3LTR) AND NOT (vsaRNA)$ [S13]

The synthesis of Tat depends on the presence of mRNA of 2 kb and 4 kb (3):

$Tat= (RNAs2kbC) OR (RNAs4kbC)$ [S14]

The synthesis of Rev depends on the synthesis of mRNA of 2 kb (3):

$$Rev= RNAs2kbC$$

[S15]

Similarly with Nef:

$Nef= RNAs2kbC$ [S16]

The synthesis of Vpr is regulated in the same way as the Tat, then:

$Vpr= (RNAs2kbC) OR (RNAs4kbC)$ S17]

Finally, the expression of p24Gag only depends of mRNA of 9 kb (3):

$p24Gag= RNAs9kbC$ [S18]

The network inputs are: TNFα and I_HMTs_.

Boolean rules:

| $TNF\alpha$ | $=$ | $input$ |
| --- | --- | --- |
| $I_{HMTS}$ | $=$ | $input$ |
| $NF-\kappa B$ | $=$ | $\left( TNF\alpha\right) OR \left( Tat \right) OR \left( Vpr \right) OR (Nef)$ |
| $HMTs$ | $=$ | $\left( I_{HMTS} \right) OR \left( asRNA \right)$ |
| $p'5LTR$ | $=$ | $(NFkB) AND NOT (HMTs)$ |
| $p'3LTR$ | $=$ | $(NFkB) AND NOT (HMTs)$ |
| $RNAs9kbN$ | $=$ | $p'5LTR$ |
| $vsiRNA$ | $=$ | $\left( p'5LTR \right) AND NOT (Tat)$ |
| $vsaRNA$ | $=$ | $(p'5LTR)$ |
| $RNAs4kbN$ | $=$ | $RNAs9kbN$ |
| $RNAs2kbN$ | $=$ | $RNAs4kbN$ |
| $RNAs2kbC$ | $=$ | $(RNAs2kbN) AND NOT (vsiRNA)$ |
| $RNAs4kbC$ | $=$ | $(RNAs4kbN) AND (Rev) AND NOT (vsiRNA)$ |
| $RNAs9kbC$ | $=$ | $(RNAs9kbN) AND (Rev) AND NOT (vsiRNA)$ |
| $asRNA$ | $=$ | $(p'3LTR) AND NOT (vsaRNA)$ |
| $Tat$ | $=$ | $(RNAs2kbC) OR (RNAs4kbC)$ |
| $Rev$ | $=$ | $RNAs2kbC$ |
| $Nef$ | $=$ | $RNAs2kbC$ |
| $Vpr$ | $=$ | $(RNAs2kbC) OR (RNAs4kbC)$ |
| $p24Gag$ | $=$ | $RNAs9kbC$ |

Details of the Boolean model analysis

*Update schemes*

The Boolean networks are classified according to their update scheme. In a general, there are three different update schemes to know: a) synchronous update, b) semi­ synchronous update and c) asynchronous update. For more specific details about this classification see the reference (31).

*Update order of the synchronous scheme*

On the original work of Kauffman, the nodes update in a synchronous way. This means that all the nodes in the network are updated simultaneously. Despite in a biological scenario this model is not realistic; it has been successfully used to model biological networks.

*Update order of the semi-synchronous scheme*

The semi-synchronous scheme only updates synchronously a set of nodes at the same time $t$. On this procedure the set of nodes to be updated is selected in a similar way to a cascade: the entries of the network are updated firstly, and then its corresponding outputs are updated. This process continues until update a set of nodes that lack of outputs. The HIV-1 network has three inputs: TNF and intracellular inducers of HMTs and HATs (I_HMTs_, I_HATs_), both inputs are updated simultaneously and then, other set of nodes are updated. It takes 9 time steps to update the entire network of the HIV-1. We repeat this process 200 times in order to reach the network attractors. We present the full update cascade in Table A.

**Table A. Update sequence of the semi-synchronous scheme**

| **Update step** | **Updated nodes** |
| --- | --- |
| t = 1/9 | {TNF, I_HMTS_}* |
| t = 2/9 | {NF-κB, HMTs}‡ |
| t = 3/9 | {p’5LTR, p’3LTR} |
| t = 4/9 | {RNAs9kbN, vsiRNA, vsaRNA, asRNA} |
| t = 5/9 | {HMTs, RNAs4kbN, RNAs2kbC, RNAs4kbC, RNAs9kbC, asRNA} |
| t = 6/9 | {HMTs, p’5LTR, p’3LTR, RNAs2kbN, RNAs4kbC, Tat, Rev, Nef, Vpr, p24Gag} |
| t = 7/9 | {NF-κB, p’5LTR, p’3LTR, RNAs9kbN, vsiRNA, vsaRNA, RNAs2kbC, RNAs4kbC, RNAs9kbC, asRNA, Tat, Vpr} |
| t = 8/9 | {NF-κB, HMTs, p’5LTR, p’3LTR, RNAs9kbN, vsiRNA, vsaRNA, RNAs4kbN, RNAs2kbC, RNAs4kbC, RNAs9kbC, asRNA, Tat, Rev, Nef, Vpr, p24Gag} |
| t = 1 | {NF-κB, HMTs, p’5LTR, p’3LTR, RNAs9kbN, vsiRNA, vsaRNA, RNAs4kbN, RNAs2kbN, RNAs2kbC, RNAs4kbC, RNAs9kbC, asRNA, Tat, Rev, Nef, Vpr, p24Gag} |
| *Network inputs. ‡ Each set of nodes is synchronously updated. | |

*Update order of the asynchronous scheme*

The asynchronous update is based on the principle that it is not possible that the nodes of a biological network are simultaneously updated, because genetic expression is a succession of events. It means that the change of expression of a specific node depends exclusively of its inputs. This is clearly exemplified on the signaling pathways, where there is a node defined as the input, and when it is activated, other nodes are updated one by one, like a cascade. For instance the hypothetical pathway:

$$\sigma_{n_{1}}\to\sigma_{n_{2}}\to\cdots\to\sigma_{n_{k}}$$

Here the state of the node $\sigma_{n_{i}}$only depends of the state of the previous node $\sigma_{n_{i-1}}$; thus the asynchronous update scheme pretends to reproduce the dynamics of hierarchical intracellular processes.

In general the asynchronous schemes require knowing all update times of biological networks; however sometimes it is not easy to elucidate them. We solve this problem by using the update order generated from the semi-synchronous scheme. The semi-synchronous scheme partially shows the succession of events required for expressing the provirus genome as well as the update order for 9 sets of nodes, and then we use biological considerations to determine which node may be updated first within such sets. The full update cascade is formed by 65 time steps (Table B), and we repeat this process 200 times in order to reach the network attractors.

**Table B. Update sequence of the asynchronous scheme**

| **Update step** | **Updated nodes** | **Biological criteria** |
| --- | --- | --- |
| t = 1/65 | TNF | Inflammation signaling strongly modifies the cell(13). |
| t = 2/65 | I_HMTS_ | This is the network input. |
| t = 3/65 | NF-κB | NF-κB is the main target of TNF signaling (26). |
| t = 4/65 | HMTs | HMTs is activated directly by I_HMTS_ |
| t = 5/65 | p’5LTR | This promoter is more active than the 3’LTR (16). |
| t = 6/65 | p3LTR | This promoter is less active than the 5’LTR (16). |
| t = 7/65 | RNAs9kbN | This is the first product of 5’LTR promoter (3). |
| t = 8/65 | vsiRNA | Inner sections of the genomic RNA (9kb) have vsiRNA (6) |
| t = 9/65 | vsaRNA | Inner sections of the genomic RNA (9kb) have vsaRNA(4). |
| t = 10/65 | asRNA | This is the main product of the 3’LTR promoter (16). |
| t = 11/65 | HMTs | This enzyme is activated by asRNAs(16). |
| t = 12/65 | RNAs4kbN | This is a splicing product of RNA9kbN (3). |
| t = 13/65 | {RNAs4kbC, RNAs9kbC}‡ | Rev may transport both RNAs at the same time (3) |
| t = 14/65 | asRNA | This is the main product of the 3’LTR promoter (16). |
| t = 15/65 | HMTs | This enzyme is activated by asRNAs(16). |
| t = 16/65 | {p’5LTR, p3LTR}‡ | Both promoters are repressed by the HMTs (16). |
| t = 17/65 | RNAs2kbN | Before activation, this is the most abundant RNA (3). |
| t = 18/65 | RNAs4kbC | These RNAs remain for long time in the cytoplasm (27). |
| t = 19/65 | Nef | Nef is the first early protein to be detected (28). |
| t = 20/65 | Rev | Rev is the second early protein to be detected (28). |
| t = 21/65 | {Tat, Vpr}‡ | Tat and Vpr are the least abundant of the viral proteins (28). |
| t = 22/65 | p24Gag | Late proteins are detected after early proteins (28). |
| t = 23/65 | NF-κB | NF-κB is activated by Tat, Vpr and Nef(13,14,29) |
| t = 24/65 | p’5LTR | This promoter is more active than the 3’LTR (16). |
| t = 25/65 | p3LTR | This promoter is less active than the 5’LTR (16). |
| t = 26/65 | RNAs9kbN | This is the first product of 5’LTR promoter (3). |
| t = 27/65 | vsiRNA | Inner sections of the genomic RNA (9kb) have vsiRNA (6) |
| t = 28/65 | vsaRNA | Inner sections of the genomic RNA (9kb) have vsaRNA(4). |
| t = 29/65 | RNAs4kbN | This is a splicing product of RNA9kbN (3). |
| t = 30/65 | RNAs2kbC | The transport of this RNA does not depend on Rev (3) |
| t = 31/65 | RNAs4kbC | These RNAs remain for long time in the cytoplasm (27) |
| t = 32/65 | RNAs9kbC | These RNAs remain for long time in the cytoplasm (27). |
| t = 33/65 | asRNA | This is the main product of the 3’LTR promoter (16). |
| t = 34/65 | {Tat, Vpr}‡ | Tat and Vpr are encoded in RNAs of 2kb and 4kb (3). |
| t = 35/65 | NF-κB | NF-κB is activated by Tat and Vpr(13,29). |
| t = 36/65 | HMTs | This enzyme is activated by asRNAs(16). |
| t = 37/65 | p’5LTR | This promoter is more active than the 3’LTR (16). |
| t = 38/65 | p3LTR | This promoter is less active than the 5’LTR (16). |
| t = 39/65 | RNAs9kbN | This is the first product of 5’LTR promoter (3). |
| t = 40/65 | vsiRNA | Inner sections of the genomic RNA (9kb) have vsiRNA (6) |
| t = 41/65 | vsaRNA | Inner sections of the genomic RNA (9kb) have vsaRNA (4). |
| t = 42/65 | RNAs4kbN | This is a splicing product of RNA9kbN (3) |
| t = 43/65 | RNAs2kbC | The transport of this RNA does not depend on Rev (3) |
| t = 44/65 | {RNAs4kbC, RNAs9kbC}‡ | Rev may transport both RNAs at the same time (3). |
| t = 45/65 | asRNA | This is the main product of the 3’LTR promoter (16). |
| t = 46/65 | Nef | Nef is the first early protein to be detected (28). |
| t = 47/65 | Rev | Rev is the second early protein to be detected (28). |
| t = 48/65 | {Tat, Vpr}‡ | Tat and Vpr are the least abundant of the viral proteins (28). |
| t = 49/65 | p24Gag | Late proteins are detected after early proteins (28). |
| t = 50/65 | NF-κB | NF-κB is activated by Tat, Vpr and Nef(13,14,29) |
| t = 51/65 | HMTs | This enzyme is activated by asRNAs(16) |
| t = 52/65 | p’5LTR | This promoter is more active than the 3’LTR (16). |
| t = 53/65 | p3LTR | This promoter is less active than the 5’LTR (16). |
| t = 54/65 | RNAs9kbN | This is the first product of 5’LTR promoter (3). |
| t = 55/65 | vsiRNA | Inner sections of the genomic RNA (9kb) have vsiRNA (6) |
| t = 56/65 | vsaRNA | Inner sections of the genomic RNA (9kb) have vsaRNA(4). |
| t = 57/65 | RNAs4kbN | This is a splicing product of RNA9kbN (3). |
| t = 58/65 | RNAs2kbC | The transport of this RNA does not depend on Rev (3) |
| t = 59/65 | RNAs2kbN | Before activation, this is the most abundant RNA (3) |
| t = 60/65 | {RNAs4kbC, RNAs9kbC}‡ | Rev may transport both RNAs at the same time (3) |
| t = 61/65 | asRNA | This is the main product of the 3’LTR promoter (16). |
| t = 62/65 | Nef | Nef is the first early protein to be detected (28). |
| t = 63/65 | Rev | Rev is the second early protein to be detected (28). |
| t = 64/65 | {Tat, Vpr}‡ | Tat and Vpr are the least abundant of the viral proteins (28). |
| t = 1 | p24Gag | Late proteins are detected after early proteins (28). |
| *Network inputs. ‡ Each set of nodes is synchronously updated. | | |

*Intersection of network states*

Previously it was reported that the size of the basins of attraction may be affected by the update scheme employed to analyze a network (31). For that reason, we used the three update schemes previously described to analyze the provirus’ gene expression network. Then, we calculate the intersection of the basins of viral activation obtained with synchronous, asynchronous and semi-synchronous update schemes by determining which states are contained into the three basins. As a result of this calculus, we find out a conserved set of intracellular environments that may activate the provirus (Fig. 2B).

*Derrida mapping test*

Critical systems integrate and process information faster and efficiently than non-critical systems. They respond to stimuli in a wide range of variations and especially exhibit collective behaviors and coordinate responses. The above is a product of the existence of long-range correlations in the systems (32).

Some studies have shown that genetic networks of several organism exhibit critical dynamics (32,33). Criticality confers to genetic networks very interesting properties from the biological perspective. One of these properties is the coexistence of robustness (the invariance of the phenotypes to perturbations) and the evolvability (the ability of the organism to change and adapt to new environment). Such coexistence would be difficult to explain in absence of critical dynamics.

The Kaufmann model exhibits two dynamical regimes, Ordered and chaotic, and a phase transition between them, the so-called critical point. The characterization of these regimes is given by the behavior of the avalanche of perturbations (produced by thermal fluctuations, gene knockout or gene over expression). In the chaotic regime, small perturbations spread throughout the network over time, producing big changes in the network state. Therefore, a network operating in a chaotic regime and submerged in a noisy cellular environment would have very unstable phenotypes. In the order regime, the perturbations die out over time, preventing the network to adapt to new environmental challenges. In the critical point, the perturbations neither spread to the entire network nor disappear. They remain confined within a small fraction of genes. Additionally, at the critical point the network is resilient to random mutations and at the same time is capable of useful innovation of phenotypes. These apparently opposite properties are able to coexist with the highest probability in the critical point (34). In order to characterizes the dynamical regime, we define the normalized Hamming distance *h*(*t*) at time $t$ between two network states as:

$$h\left( t \right)= \frac{1}{N}\sum_{N=1}^{N} \left| \sigma_{n}\left( t \right)- \tilde{\sigma}_{n}(t) \right|$$

Where $\sigma_{n}(t)$ is the state of the *n*-th gene at time *t* in a trajectory starting out from a given initial condition, and $\tilde{\sigma}_{n}(t)$ is the state of the same gene in a different trajectory generated from a different initial condition. The Hamming distance *h*(*t*) can be considered as the normalized size of the avalanche of perturbations generated by differences the initial condition. The Derrida map *h*(*t +* 1) = *M*(*h*(*t*)) (35) relates the size of the avalanche at two consecutive time steps. It can be shown that *M*(*h*) is a monotonic increasing function with the property that *M*(0) = 0 (if there is no perturbation at time *t*, there is no perturbation either at time *t* + 1). Therefore, the slope *S* at the origin of *M*(*h*) is the parameter that characterizes the asymptotic value of the Hamming distance, and hence the network dynamics. *S* is called the average network sensitivity. When *S <* 1, the network is operating in the ordered regime. If *S* > 1, the network exhibits chaotic behavior. If *S* = 1, the network is in the critical regime. An intuitive definition from (36) is that *S* is the average number of genes that change their state at time *t +* 1 when a single gene is perturbate at time *t*.

*Numerical calculation of Derrida Test*

We calculate the Derrida test selecting a random state of the network, denoted by $\sum_{t}$. We apply the dynamic one time step to obtain the next state $\sum_{t+1}$. From $\sum_{t}$, we choose randomly a fraction $h$ of genes, then we change their state and this generates another network state $\tilde{\sum}_{t}$. Then we apply the dynamic one time step to obtain $\tilde{\sum}_{t+1}$. We evaluated the Hamming distance between $\sum_{t+1}$ and $\tilde{\sum}_{t+1}$. Each point of the graph (Figure 2C) is an average of 10,000 pairs of states $\sum_{t+1}$ and $\tilde{\sum}_{t+1}$.

According to the above, we calculate the Derrida Map for the provirus and obtain the value of *average* *network sensitivity* *S*. As seen in Figure 2C this value is very close to criticality. This may explain the robustness and adaptability of the provirus. Interestingly, previous works have reported criticality in bacteria and eukaryotic organisms (32,33); and complementing such reports, we characterize by the first time criticality on a virus.

Derivation of the ODEs model

*Regulation of the activity of LTR promoters*

Gene transcription is a complex process that requires chromatin remodeling, and the binding of several transcription factors (TFs) and RNA polymerase II (RNAP) to the promoter region. The first point for the regulation of the expression of a gene is activating TFs availability. In the case of HIV-1, there are many TFs that either activate or repress viral gene expression. The most notable is the nuclear factor kappa B (NF-κB), which is present in many immune cells (13,29,37). Several DNA regulatory sequences facilitate the binding of more TFs once they are positioned in the promoter (i.e., cooperativeness); however, recent evidence indicates that this is not the case of the promoters regulated by NF-κB enhancers (37). In this form, we assumed that only one molecule of free NF-κB (NF-κB*) is required to activate transcription of LTR promoters, in other words:

On the other hand, chromatin remodeling can enhance or avoid gene expression. Histone acetylation relaxes chromatin and enhances gene expression, while histone methylation compacts chromatin and produces the opposite effect (1,11,38). In order to include these facts in our model of RNA transcription, we considered that histone acetylation increases TFs affinity for its DNA binding sequences, allowing their union to the target sequences with higher probability. We assumed that histone methylation has the opposite effect because they promote DNA compaction, and the TFs cannot bind to their target sequences (1). Applying these considerations to the regulation of LTR promoters, we can say that:

All viral RNAs transcribed by the 5’LTR promoter have a special RNA structure known as trans-activator response element (TAR). This RNA loop interacts with the viral trans-activator of transcription protein (Tat) and the positive transcription elongation factor (P-TEFb) to form a complex that hyperphosphorilates RNAP. In consequence, TAR increases the transcription of full genomic RNAs of 9 kb (mRNA9kb) (8). Adding such interaction to 5’LTR regulation, we have:

Similarly, for 3’LTR promoter we obtain:

Then, the 5’LTR promoter activity could be represented with a modified Michaelis Menten equation as:

$$\frac{d}{dt}\left[5'LTR*\right]=k_{b}\left(1+k_{ac}\left[HATs\right]+k_{tar}\left[Tat\right]\right)\left[RNAP\right]\left[NF\kappa B\right]-k_{d}\left(1+k_{me}\left[HMTs\right]\right)\left[5'LTR*\right]$$

In the same way, 3’LTR promoter activity is given by:

$$\frac{d}{dt}\left[3'LTR*\right]=k_{b}\left(1+k_{ac}\left[HATs\right]\right)\left[RNAP\right]\left[NF\kappa B\right]-k_{d}\left(1+k_{me}\left[HMTs\right]\right)\left[3'LTR*\right]$$

Assuming that the concentration of RNAP is constant within the cell, i.e. $\left[RNAP\right]_{T}=\left[RNAP\right]+\left[3'LTR\right]+\left[5'LTR\right]$we obtain:

$\frac{d}{dt}\left[5'LTR*\right]=k_{b}\left(1+k_{ac}\left[HATs\right]+k_{tar}\left[Tat\right]\right)\left(\left[RNAP\right]_{T}-\left[3'LTR\right]-\left[5'LTR\right]\right)\left[NF\kappa B\right]-k_{d}\left(1+k_{me}\left[HMTs\right]\right)\left[5'LTR*\right]$ [S20]

$\frac{d}{dt}\left[3'LTR*\right]=k_{b}\left(1+k_{ac}\left[HATs\right]\right)\left(\left[RNAP\right]_{T}-\left[3'LTR\right]-\left[5'LTR\right]\right)\left[NF\kappa B\right]-k_{d}\left(1+k_{me}\left[HMTs\right]\right)\left[3'LTR*\right]$ [S21]

*Regulation of viral transcription*

Once the 5’LTR promoter is active, viral RNAs of 9 KB (RNA9kb) are transcribed with a rate of synthesis *a*_1_. Then they could be spliced with a rate *s_1_*, and transported to the cytoplasm either by the Rev-RRE interaction with a rate *k_RRE_*, or by an independent mechanism (39) (parameter *τ*). Finally, this transcript is degraded with a rate *δ_1_*.

The interactions that regulate the levels of non-spliced RNA of 9 KB concentration can be modeled by a mass-balance equation as follows:

$\frac{d}{dt}\left[mRNA_{9kb}\right]=a_{1}\left[p5LTR*\right]-\left(s_{1}+\tau+k_{RRE}\left[\mathrm{Re}v\right]+\delta_{1}\right)\left[mRNA_{9kb}\right]$ [S22]

The 5’LTR promoter also generates several truncated RNAs with a rate α_2_; that are processed to viral small RNAs. Many of them can repress cell functions as well as viral RNAs in the cytoplasm (6,40,41). Recently it was shown that Tat is able to interfere with the synthesis of such RNAs once the virus starts its replication (42) (with a rate r_1_.).

In mathematical terms, the temporal variation of the concentration of viral small interfering RNAs (vsiRNA) is given by:

$\frac{d}{dt}\left[vsiRNA\right]=a_{2}\left[RNA_{9kb}\right]-\left(\delta_{2}+r_{1}\left[Tat\right]\right)\left[vsiRNA\right]$ [S23]

Recently has been reported a new type of viral small RNAs derived from the Pol gene that are able to activate viral transcription (4). Its concentration only depends of the irreversible fluxes of production and degradation as follows:

Then, the viral small activating RNAs (vsaRNA) concentration is given by the equation:

$\frac{d}{dt}\left[vsaRNA\right]=a_{3}\left[RNA_{9kb}\right]-\delta_{3}\left[vsaRNA\right]$ [S24]

On the other hand, the *nef*-3’LTR region has an antisense promoter that transcribes an antisense long non-coding RNA (asRNA) which interacts with the histone methyl-transferases in order to selectively repress viral genome expression (16). In a physiological context, the function of cellular asRNAs can be interfered by interactions with small RNAs that target promoter regions (18). The recent characterization of long asRNA (16) as well as small activating RNAs (4) encoded by the HIV-1 genome, assuming that this regulatory mechanism exists to regulate provirus gene expression. The concentration of inhibitory asRNAs depends on its synthesis (*a*_4_) and degradation (*δ_4_*) rates, as well on the suppression of repressor activity mediated by vsaRNA (r_2_), in other words:

Thus, the mass-balance equation for these interactions is:

$\frac{d}{dt}\left[asRNA\right]=a_{4}\left[p3LTR*\right]-\left(r_{2}\left[vsaRNA\right]+\delta_{4}\right)\left[asRNA\right]$ [S25]

When nuclear RNA9kb is fully transcribed, it is spliced into partially processed RNAs of 4 kb (RNA4kb) or totally processed RNAs of 2 kb (RNA2kb) (3). Nuclear RNA4kb can also be transported to the cytoplasm either by the Rev-RRE interaction with a rate *k_RRE_*, or by an independent mechanism *τ*, or it can be degraded with a rate *δ_5_*. These temporal variations can be represented as follows:

These irreversible fluxes can be described in the equation:

$\frac{d}{dt}\left[mRNA_{4kb}\right]=s_{1}\left[RNA_{9kb}\right]-\left(s_{2}+\tau+k_{RRE}\left[\mathrm{Re}v\right]+\delta_{5}\right)\left[mRNA_{4kb}\right]$ [S26]

On the other hand, fully spliced viral mRNAs of 2 kb can abandon the nucleus without Rev-RRE interaction with a rate *k_exp_*, and can be degraded with a rate *δ_6_*, i. e.

Then, the mass-balance equation for the concentration of nuclear RNA2kb is given by:

$\frac{d}{dt}\left[mRNA_{2kb}\right]=s_{2}\left[mRNA_{4kb}\right]-\left(k_{\exp}+\delta_{6}\right)\left[mRNA_{2kb}\right]$ [S27]

Once viral RNAs of 2 KB, 4 KB and 9 KB are in the cytoplasm they can be silenced by the RISC using vsiRNA (41,42) with a rate *r_3_*, and can be degraded with a rate *δ_i, i = 7, 8, 9_* . In other words:

$\frac{d}{dt}\left[mRNA_{2kb}^{C}\right]=k_{\exp}\left[mRNA_{2kb}\right]-\left(r_{3}\left[vsiRNA\right]+\delta_{7}\right)\left[mRNA_{2kb}^{C}\right]$ [S28]

$\frac{d}{dt}\left[mRNA_{4kb}^{C}\right]=\left(\tau+k_{RRE}\left[\mathrm{Re}v\right]\right)\left[mRNA_{4kb}\right]-\left(r_{3}\left[vsiRNA\right]+\delta_{8}\right)\left[mRNA_{4kb}^{C}\right]$ [S29]

$\frac{d}{dt}\left[mRNA_{9kb}^{C}\right]=\left(\tau+k_{RRE}\left[\mathrm{Re}v\right]\right)\left[mRNA_{9kb}\right]-\left(r_{3}\left[vsiRNA\right]+\delta_{9}\right)\left[mRNA_{9kb}^{C}\right]$ [S30]

*Regulation of viral translation*

When viral RNAs are in the cytoplasm, they are translated into viral proteins. To be precise, the early proteins Tat, Rev, Nef and Vpr are encoded in RNA2kb (3). Moreover, there are isoforms of Tat and Vpr which are encoded by RNA4kb (3). Thus, the irreversible fluxes that regulate the concentration of Tat are given by:

Then, the mass-balance equation for the concentration of Tat is:

$\frac{d}{dt}\left[Tat\right]=a_{5}\left[mRNA_{2kb}^{C}\right]+a_{6}\left[mRNA_{4kb}^{C}\right]-\delta_{10}\left[Tat\right]$ [S31]

For Rev, the fluxes that regulate its intracellular levels are:

However, intracellular levels of Rev do not change because of its effector functions of viral mRNA transportation, because this protein is not degraded in the transporting process. Then, we simplify the fluxes as follows:

Therefore, the mass-balance equation for Rev intracellular levels is:

$\frac{d}{dt}\left[\mathrm{Re}v\right]=a_{7}\left[mRNA_{2kb}^{C}\right]-\delta_{11}\left[\mathrm{Re}v\right]$ [S32]

Applying the same assumption of simplified fluxes to Nef concentration, we considered:

Then, the equation of Nef concentration is given by:

$\frac{d}{dt}\left[Nef\right]=a_{8}\left[mRNA_{2kb}^{C}\right]-\delta_{12}\left[Nef\right]$ [S33]

Following the same logic of Tat expression, the fluxes that rule the intracellular levels of Vpr are:

Thus, the mass-balance equation for intracellular Vpr is:

$\frac{d}{dt}\left[Vpr\right]=a_{9}\left[mRNA_{2kb}^{C}\right]+a_{10}\left[mRNA_{4kb}^{C}\right]-\delta_{13}\left[Vpr\right]$ [S34]

Finally, the late proteins Gag, Pol, Env, Vpu and Vif are encoded by RNA9kb and RNA4kb respectively (3). In our model we used p24Gag as the output of the system because it is the most abundant late protein of the HIV-1. Then, using the assumption of simplified fluxes, the equation of p24Gag is given by:

$\frac{d}{dt}\left[p24^{Gag}\right]=a_{11}\left[mRNA_{9kb}^{C}\right]-\delta_{14}\left[p24^{Gag}\right]$ [S35]

*Regulation of viral interactions with the host*

It is known that HIV-1 has many interactions, which down regulate its host; the most remarkable are the NF-κB dependent feedback loops. NF-κB is activated by TNF, although it was demonstrated recently that can be activated also by the viral proteins Tat, Nef and Vpr (13–15). Here, we consider only the concentration of active NF-κB (NF-κB*) instead of total concentration, in other words:

$\frac{d}{dt}\left[NF\kappa B*\right]=k_{1}\left(\left[NF\kappa B\right]_{T}-\left[NF\kappa B*\right]\right)\left(k_{0}\left[TNF\alpha\right]+k_{2}\left[Tat\right]+k_{3}\left[Nef\right]+k_{4}\left[Vpr\right]\right)-k_{-1}\left[NF\kappa B*\right]$ [S36]

where: $\left[NF\kappa B\right]_{T}=\left[NF\kappa B\right]+\left[NF\kappa B*\right]$.

The viral genome interacts with epigenetic factors like HATs and HMTs as follows: Tat (11) and Vpr (12) recruit HATs to LTR promoters.

Thus, the concentration of active HATs (HATs*) is given by:

$\frac{d}{dt}\left[HATs*\right]=k_{5}\left(\left[HATs\right]_{T}-\left[HATs*\right]\right)\left(I_{HATs}+k_{6}\left[Tat\right]+k_{7}\left[Vpr\right]\right)-k_{-5}\left[HATs*\right]$ [S37]

where: $\left[HATs\right]_{T}=\left[HATs\right]+\left[HATs*\right]$

Regarding HMTs, it has been demonstrated recently that asRNA positions HMTs on LTR promoter to silence it (16). Thus, the concentration of active HMTs is expressed by:

$\frac{d}{dt}\left[HMTs*\right]=k_{8}\left(\left[HMTs\right]_{T}-\left[HMTs*\right]\right)\left(I_{HMTs}+k_{9}\left[asRNA\right]\right)-k_{-8}\left[HMTs*\right]$ [S38]

where: $\left[HMTs\right]_{T}=\left[HMTs\right]+\left[HMTs*\right]$

*Input functions*

Finally, we summarize all possible inputs of the viral network assuming that: 1) viral transcription is mainly regulated by immune activation mediated by TNF; 2) HMTs and HATs are controlled independently by several factors; 3) the activity of TNF, HATs and HMTs can be turned off by complex molecular mechanisms; 4) activation and repression could occur in a non-specified time interval. Therefore, the stimulation of TNF, the inducers of HATs (I_HATs_) and the inducers of HMTs (I_HMTs_) are described by:

$TNF\alpha=\left\{\begin{matrix} 1,\begin{matrix} & & \end{matrix}t\in T_{1} \\ 0,\begin{matrix} & & \end{matrix}t\notin T_{1} \end{matrix}\right.$ [S39]

$I_{HATs}=\left\{\begin{matrix} 1,\begin{matrix} & & \end{matrix}t\in T_{2} \\ 0,\begin{matrix} & & \end{matrix}t\notin T_{2} \end{matrix}\right.$ [S40]

$I_{HMTs}=\left\{\begin{matrix} 1,\begin{matrix} & & \end{matrix}t\in T_{3} \\ 0,\begin{matrix} & & \end{matrix}t\notin T_{3} \end{matrix}\right.$ [S41]

Where:

$$T_{1}=\left[\text{25,80}\right]\cup\left[\text{525,530}\right]\cup\left[\text{825,830}\right]$$

$$T_{2}=\left[\text{10,50}\right]\cup\left[\text{170,200}\right]\cup\left[\text{470,500}\right]$$

$$T_{3}=\left[\text{57,187}\right]\cup\left[\text{507,577}\right]\cup\left[\text{707,777}\right]\cup\left[\text{957,1000}\right]$$

*Notes*: The value of initial conditions in all simulations was zero for all variables of the system. The full set of parameters of this model is shown in Table C.

**Table C. Parameters of the ODEs model and their source**

| **Symbol** | **Value** | **Meaning** | **Reference** |
| --- | --- | --- | --- |
| *k_5_*  *k_6_*  *k_7_*  *k_-5_* | 0.1 min^-1^  2 a.u.c.^-1^  2 a.u.c.^-1^  0.2 min^-1^ | Activation rate of HATs  Activation due to Tat interactions  Activation due to Vpr interactions  Deactivation of HATs | *  *  *  * |
| *k_8_*  *k_9_*  *k_-8_* | 0.1 min^-1^  0.6 a.u.c.^-1^  0.1 min^-1^ | Activation of HMTs  Activation due to asRNA  Deactivation of HMTs | *  (16)  * |
| *k_0_*  *k*_1_  *k*_2_  *k*_3_  *k*_4_  *k*_-1_ | 1 a.u.c.^-1^  0.1 min^-1^  2 a.u.c.^-1^  2 a.u.c.^-1^  2 a.u.c.^-1^  0.3 min^-1^ | Stimulation due to TNFα  Availability of NFκB  Activation due to Tat interactions  Activation due to Nef interactions  Activation due to Vpr interactions  Repression of NF-κB | *  *  (22)  (14)  (12)  * |
| *k_b_*  *k_ac_*  *k_TAR_*  *k_d_*  *k_me_* | 0.102 min^-1^  29.4 a.u.c.^-1^  147 a.u.c.^-1^  0.2 min^-1^  100 a.u.c.^-1^ | Opening rate of 5'LTR promoter  Rate of acetylation of LTR promoter  Rate of transactivation mediated by Tat-TAR interactions  Closing rate of 5'LTR promoter  Rate of methylation of LTR promoter | *  *  (43)  *  *  (44) |
| *a*_1_  *s*_1_  *τ*  *k_REE_*  *δ*_1_ | 2 min^-1^  0.59 min^-1^  0.1 min^-1^  0.1 (a.u.c.*min)^-1^  0.01 min^-1^ | Rate of viral RNA synthesis  Splicing rate  Rate of Rev-independent RNA transport to the cytoplasm  Rate of Rev-dependent RNA transport to the cytoplasm  Degradation rate of RNA9kb in the nucleus | *  *  *  *  * |
| *a*_2_  *δ*_2_  *r*_1_ | 0.1 min^-1^  0.3 min^-1^  10 (a.u.c.*min) ^-1^ | Rate of vsiRNA synthesis  Degradation rate of vsiRNA  Repression mediated by Tat | *  *  * |
| *a*_3_  *δ*_3_ | 0.05 min^-1^  0.3 min^-1^ | Rate of vsaRNA synthesis  Degradation rate of vsaRNA | *  * |
| *s*_1_  *s*_2_  *τ*  *k_REE_*  *δ*_5_ | 0.2 min^-1^  0.4 min^-1^  0.1 min^-1^  0.1 (a.u.c.*min) ^-1^  0.1 min^-1^ | Splicing rate  Splicing rate  Rate of Rev-independent RNA transport to the cytoplasm  Rate of Rev-dependent RNA transport to the cytoplasm  Degradation rate of RNA4kb in the nucleus | *  *  *  *  * |
| *s*_2_  *k_exp_*  *δ*_6_ | 0.1 min^-1^  0.5 min^-1^  0.01 min^-1^ | Splicing rate  Rate of RNA transport to the cytoplasm  Degradation rate of RNA2kb in the nucleus | *  *  * |
| *k_exp_*  *r*_3_  *δ*_7_ | 0.5 min^-1^  2 (a.u.c.*min)^-1^  0.1 min^-1^ | Rate of RNA transport to the cytoplasm  Inhibition mediated by vsiRNA  Degradation rate of RNA2kb in the cytoplasm | *  *  * |
| *Τ*  *k_REE_*  *δ*_8_  *r*_3_ | 0.1 min^-1^  0.1(a.u.c.*min) ^-1^  0.5 min^-1^  2 (a.u.c.*min)^-1^ | Rate of Rev-independent RNA transport to the cytoplasm  Rate of Rev-dependent RNA transport to the cytoplasm  Degradation rate of RNA4kb in the cytoplasm  Inhibition mediated by vsiRNA | *  *  *  * |
| *Τ*  *k_REE_*  *δ*_9_  *r*_3_ | 0.1 min^-1^  0.1 (a.u.c.*min) ^-1^  0.5 min^-1^  2 (a.u.c.*min)^-1^ | Rate of Rev-independent RNA transport to the cytoplasm  Rate of Rev-dependent RNA transport to the cytoplasm  Degradation rate of RNA9kb in the cytoplasm  Inhibition mediated by vsiRNA | *  *  *  * |
| *k_b_*  *k_ac_*  *k_d_*  *k_me_* | 0.102 min^-1^  29.4 a.u.c.^-1^  0.2 min^-1^  100 a.u.c.^-1^ | Opening rate of 3'LTR promoter  Rate of acetylation of LTR promoter  Closing rate of 3'LTR promoter  Rate of methylation of LTR promoter | *  *  *  (44) |
| *a*_4_  *r*_2_  *δ*_4_ | 3 min^-1^  2 (a.u.c.*min) ^-1^  0.5 min^-1^ | Synthesis rate of asRNA  Interference mediated by vsaRNA  Degradation rate of asRNA | *  *  * |
| *a*_5_  *a*_6_  *δ*_10_ | 0.2 min^-1^  0.1 min^-1^  0.1 min^-1^ | RNA2kb-dependent synthesis of Tat  RNA4kb-dependent synthesis of Tat  Degradation rate of Tat | *  *  (45) |
| *a*_7_  *δ*_11_ | 0.5 min^-1^  0.1 min^-1^ | RNA2kb-dependent synthesis of Rev  Degradation rate of Rev | *  (46) |
| *a*_8_  *δ*_12_ | 2 min^-1^  0.1 min^-1^ | RNA2kb-dependent synthesis of Nef  Degradation rate of Nef | *  * |
| *a*_9_  *a*_10_  *δ*_13_ | 0.2 min^-1^  0.1 min^-1^  0.1 min^-1^ | RNA2kb-dependent synthesis of Vpr  RNA4kb-dependent synthesis of Vpr  Degradation rate of Vpr | *  *  (47) |
| *a*_11_  *δ*_14_ | 10 min^-1^  0.5 min^-1^ | RNA9kb-dependent synthesis and processing of p24Gag  Degradation rate of p24Gag | *  (48) |

*These parameters are set at arbitrary values used in this work.

Details of the ODEs model analysis

*Stability analysis*

In order to find the equilibrium or fixed points of the model and explore their stability, we used the perturbation method described in this section.

Consider a system of *n*-independent variables in which the state of the system at time *t* is determined by the set of variables$\left\{x_{1}\left(t\right),x_{2}\left(t\right),...,x_{n}\left(t\right)\right\}$. The respective dynamical system is the set of nonlinear differential equations , subject to the initial conditions$\left\{x_{1o}\left(0\right),x_{2o}\left(0\right),...,x_{no}\left(0\right)\right\}$. The set of variables $\left\{x_{1}\left(t\right),x_{2}\left(t\right),...,x_{n}\left(t\right)\right\}$defines the phase space of the dynamical system in which the motion of the system occurs. As time goes on, the point $\mathbf{x}\left(t\right)=\left\langle x_{1}\left(t\right),x_{2}\left(t\right),...,x_{n}\left(t\right)\right\rangle$moves along the phase space and defines a curve o trajectory of the system for each initial condition. The objective is to analyze the dynamical system in order to know the complete set of trajectories of the system in the phase space (the phase portrait), and to discern the behavior of these trajectories in the neighborhood of the equilibrium o fixed points of the dynamical system.

Equilibrium or fixed points are the points of the phase space in which all the derivates becomes zero. If fluctuations *δx_1_,* *δx_2_,…,* *δx_n_* perturb the fixed point **x**^o^, the system is displaced to a new state$\delta\mathbf{x}^{o}=\left(x_{1}^{o}+\delta x_{1},x_{2}^{o}+\delta x_{2},...,x_{n}^{o}+\delta x_{n}\right)$, and the trajectory that emerges from this point of the phase space can either bring closer to the original equilibrium or go far away from it. If the trajectory tends to the fixed point in an asymptotic form, then **x**^o^ is stable, and it is an attractor. If the trajectory moves away from the fixed point, then **x**^o^ is unstable, and it is a repeller.

The dynamical behavior of the fluctuations determines the stability of the system, and is essential to establish a form to analyze the evolution of the fluctuation along time. In order to achieve this goal, it is necessary to assume that the behavior of the fluctuations in the neighborhood of the fixed point is linear, i.e., the nonlinear terms can be neglected, obtaining the Jacobian of the linearized dynamical system as:

$$\mathbf{J}\left(\mathbf{x}^{o}\right)=\left[\begin{matrix} \frac{\partial f_{1}}{\partial x_{1}} & \cdots& \frac{\partial f_{1}}{\partial x_{n}} \\ \vdots& \ddots& \vdots\\ \frac{\partial f_{n}}{\partial x_{1}} & \cdots& \frac{\partial f_{n}}{\partial x_{n}} \end{matrix}\right]_{\mathbf{x}^{o}}$$

The evolution of the trajectories in the phase space is settled on by the *n* roots of the characteristic equation of the Jacobian: $\det\left(\mathbf{J}\left(\mathbf{x}^{o}\right)-\lambda\mathbf{I}\right)=0$, where λ is a number to be determined and **I** is the identity matrix. The characteristic equation has *n* roots, known as eigenvalues, that can be real, imaginary or complex numbers. In the general case of an *n*-dimensional dynamical system there are *n* eigenvalues, and if all of them are negative real numbers or are complex numbers with negative real part the fixed point is an attractor. Otherwise, if at least one eigenvalue is a positive real number or a complex number with a positive real part, the fixed point is unstable.

In the particular case of a dynamical system with only real negative eigenvalues, the entire trajectories move toward the fixed point$\mathbf{x}^{o}$and the dynamical system has a stable node at this point. If there is a combination of real positive eigenvalues with real negative eigenvalues, the dynamical system has a saddle point at **x**^o^.

We used MATLAB to calculate the fixed points of our ODEs model (S2 Table). Also we calculate the stability of each equilibrium point and their associated eigenvalues are shown in Table D.

**Table D. Stability of equilibrium points of the HIV ODEs model**

| **Eigenvalue** | **Latency equilibrium** | **Activation equilibrium** |
| --- | --- | --- |
| λ_1_ | -0.2 | -0.5 |
| λ_2_ | -0.1 | -2.6118 |
| λ_3_ | -0.3 | -0.6403 |
| λ_4_ | -0.3 | -6.9333 |
| λ_5_ | -0.5 | -5.5864 |
| λ_6_ | -0.1 | -2.4846 |
| λ_7_ | -0.1 | -2.2840 + 0.1980i |
| λ_8_ | -0.5 | -2.2840 - 0.1980i |
| λ_9_ | -0.5 | -0.7479 + 0.0521i |
| λ_10_ | -0.6 | -0.7479 - 0.0521i |
| λ_11_ | 0.0567 | -0.5284 + 0.1182i |
| λ_12_ | -0.1004 + 0.1946i | -0.5284 - 0.1182i |
| λ_13_ | -0.1004 - 0.1946i | -0.0644 |
| λ_14_ | -0.6505 | -0.1002 + 0.0118i |
| λ_15_ | -0.4527 + 0.1593i | -0.1002 - 0.0118i |
| λ_16_ | -0.4527 - 0.1593i | -0.3137 |
| λ_17_ | -0.5 | -0.2766 |
| λ_18_ | -0.1 | -0.1 |
| λ_19_ | -0.1 | -0.1 |
| **Stability** | Re(λ) > 0 | Unstable | Re(λ) < 0 | Stable |

Supplementary References

1. Groen JN, Morris K V. Chromatin, non-coding RNAs, and the expression of HIV. Viruses. 2013;5(7):1633–45.

2. Siliciano RF, Greene WC. HIV latency. Cold Spring Harb Perspect Med. 2011;1(1):a007096.

3. Jacquenet S, Decimo D, Muriaux D, Darlix J-L. Dual effect of the SR proteins ASF/SF2, SC35 and 9G8 on HIV-1 RNA splicing and virion production. Retrovirology. 2005;2:33.

4. Zhang Y, Fan M, Geng G, Liu B, Huang Z, Luo H, et al. A novel HIV-1-encoded microRNA enhances its viral replication by targeting the TATA box region. Retrovirology. 2014;11(1):23.

5. Suzuki K, Ahlenstiel C, Marks K, Kelleher AD. Promoter Targeting RNAs: Unexpected Contributors to the Control of HIV-1 Transcription. Mol Ther Nucleic Acids. 2015;4:e222.

6. Bennasser Y, Le S-Y, Benkirane M, Jeang K-T. Evidence that HIV-1 Encodes an siRNA and a Suppressor of RNA Silencing. Immunity. 2005;22(5):607–19.

7. Robert-Guroff M, Popovic M, Gartner S, Markham P, Gallo RC, Reitz MS. Structure and expression of tat-, rev-, and nef-specific transcripts of human immunodeficiency virus type 1 in infected lymphocytes and macrophages. J Virol. 1990;64(7):3391–8.

8. D’Orso I, Jang GM, Pastuszak a. W, Faust TB, Quezada E, Booth DS, et al. A transition step during assembly of HIV Tat:P-TEFb transcription complexes and transfer to TAR RNA. Mol Cell Biol. 2012;32(23):4780–93.

9. Felber BK, Drysdale CM, Pavlakis GN. Feedback regulation of human immunodeficiency virus type 1 expression by the Rev protein. J Virol. 1990;64(8):3734–41.

10. Swingler S, Brichacek B, Jacque J-M, Ulich C, Zhou J, Stevenson M. HIV-1 Nef intersects the macrophage CD40L signalling pathway to promote resting-cell infection. Nature. 2003;424(6945):213–9.

11. Agbottah E, Deng L, Dannenberg LO, Pumfery A, Kashanchi F. Effect of SWI/SNF chromatin remodeling complex on HIV-1 Tat activated transcription. Retrovirology. 2006;3:48.

12. Felzien LK, Woffendin C, Hottiger MO, Subbramanian RA, Cohen EA, Nabel GJ. HIV transcriptional activation by the accessory protein, VPR, is mediated by the p300 co-activator. Proc Natl Acad Sci U S A. 1998;95(9):5281–6.

13. Fiume G, Vecchio E, De Laurentiis A, Trimboli F, Palmieri C, Pisano A, et al. Human immunodeficiency virus-1 Tat activates NF-κB via physical interaction with IκB-α and p65. Nucleic Acids Res. 2012;40(8):3548–62.

14. Varin A, Manna SK, Quivy V, Decrion AZ, Van Lint C, Herbein G, et al. Exogenous Nef protein activates NF-κB, AP-1, and c-Jun N-terminal kinase and stimulates HIV transcription in promonocytic cells: Role in AIDS pathogenesis. J Biol Chem. 2003;278(4):2219–27.

15. Varin A, Decrion AZ, Sabbah E, Quivy V, Sire J, Van Lint C, et al. Synthetic Vpr protein activates activator protein-1, c-Jun N-terminal kinase, and NF-κB and stimulates HIV-1 transcription in promonocytic cells and primary macrophages. J Biol Chem. 2005; 280(52):42557–67.

16. Saayman S, Ackley A, Turner A-MW, Famiglietti M, Bosque A, Clemson M, et al. An HIV-encoded antisense long noncoding RNA epigenetically regulates viral transcription. Mol Ther. 2014; 22(6):1164–75.

17. Gagnon KT, Corey DR. Argonaute and the nuclear RNAs: new pathways for RNA-mediated control of gene expression. Nucleic Acid Ther. 2012; 22(1):3–16.

18. Schwartz JC, Younger ST, Nguyen N-B, Hardy DB, Monia BP, Corey DR, et al. Antisense transcripts are targets for activating small RNAs. Nat Struct Mol Biol.. 2008; 15(8):842–8.

19. Hammarskjöld ML, Heimer J, Hammarskjöld B, Sangwan I, Albert L, Rekosh D. Regulation of human immunodeficiency virus env expression by the rev gene product. J Virol. 1989; 63(5):1959–66.

20. Fun A, Wensing AMJ, Verheyen J, Nijhuis M. Human Immunodeficiency Virus Gag and protease: partners in resistance. Retrovirology. 2012;9(1):63.

21. Zhou A, Scoggin S, Gaynor RB, Williams NS. Identification of NF-kappa B-regulated genes induced by TNFalpha utilizing expression profiling and RNA interference. Oncogene. 2003;22(13):2054–64.

22. Westendorp MO, Shatrov VA, Schulze-Osthoff K, Frank R, Kraft M, Los M, et al. HIV-1 Tat potentiates TNF-induced NF-kappa B activation and cytotoxicity by altering the cellular redox state. EMBO J. European Molecular Biology Organization; 1995;14(3):546–54.

23. Jordan A, Bisgrove D, Verdin E. HIV reproducibly establishes a latent infection after acute infection of T cells in vitro. EMBO J. 2003; 22(8):1868–77.

24. Yeung ML, Bennasser Y, Watashi K, Le S-Y, Houzet L, Jeang K-T. Pyrosequencing of small non-coding RNAs in HIV-1 infected cells: evidence for the processing of a viral-cellular double-stranded RNA hybrid. Nucleic Acids Res. 2009;37(19):6575–86.

25. Kauffman SA. Metabolic stability and epigenesis in randomly constructed genetic nets. J Theor Biol. 1969;22(3):437–67.

26. Sun Z, Arendt CW, Ellmeier W, Schaeffer EM, Sunshine MJ, Gandhi L, et al. PKC-\[thetas] is required for TCR-induced NF-κB activation in mature but not immature T lymphocytes. Nature. 2000; 404(6776):402–7.

27. Lévesque K, Halvorsen M, Abrahamyan L, Chatel-Chaix L, Poupon V, Gordon H, et al. Trafficking of HIV-1 RNA is mediated by heterogeneous nuclear ribonucleoprotein A2 expression and impacts on viral assembly. Traffic. 2006;7(9):1177–93.

28. van ’t Wout AB, Lehrman GK, Mikheeva SA, O’Keeffe GC, Katze MG, Bumgarner RE, et al. Cellular Gene Expression upon Human Immunodeficiency Virus Type 1 Infection of CD4+-T-Cell Lines. J Virol. 2003; 77(2):1392–402.

29. Liu R, Lin Y, Jia R, Geng Y, Liang C, Tan J, et al. HIV-1 Vpr stimulates NF-κB and AP-1 signaling by activating TAK1. Retrovirology. 2014; 11(1):45.

30. Maudet C, Sourisce A, Dragin L, Lahouassa H, Rain J-C, Bouaziz S, et al. HIV-1 Vpr induces the degradation of ZIP and sZIP, adaptors of the NuRD chromatin remodeling complex, by hijacking DCAF1/VprBP. PLoS One. 2013; 8(10):e77320.

31. Gershenson C. Classification of Random Boolean Networks. Artif Life VIII Proc Eighth Int Conf Artif Life. 2003;1–8.

32. Balleza E, Alvarez-Buylla ER, Chaos A, Kauffman S, Shmulevich I, Aldana M. Critical dynamics in genetic regulatory networks: Examples from four kingdoms. PLoS One. 2008;3(6).

33. Nykter M, Price ND, Aldana M, Ramsey SA, Kauffman SA, Hood LE, et al. Gene expression dynamics in the macrophage exhibit criticality. Proc Natl Acad Sci U S A. 2008; 105(6):1897–900.

34. Aldana M, Balleza E, Kauffman S, Resendiz O. Robustness and evolvability in genetic regulatory networks. J Theor Biol. 2007; 245(3):433–48.

35. Derrida B, Pomeau Y. Random Networks of Automata: A Simple Annealed Approximation. Europhys Lett. 1986; 1(2):45–9.

36. Krawitz P, Shmulevich I. Basin Entropy in Boolean Network Ensembles. Phys Rev Lett. 2007; 98(15):158701.

37. Giorgetti L, Siggers T, Tiana G, Caprara G, Notarbartolo S, Corona T, et al. Noncooperative Interactions between Transcription Factors and Clustered DNA Binding Sites Enable Graded Transcriptional Responses to Environmental Inputs. Mol Cell. 2010; 37(3):418–28.

38. Reuse S, Calao M, Kabeya K, Guiguen A, Gatot J-S, Quivy V, et al. Synergistic activation of HIV-1 expression by deacetylase inhibitors and prostratin: implications for treatment of latent infection. PLoS One. 2009; 4(6):e6093.

39. Saurya S, Lichtenstein Z, Karpas A. Defective rev response element (RRE) and rev gene in HAART treated AIDS patients with discordance between viral load and CD4+ T-cell counts. J Clin Virol. 2005; 33(4):324–7.

40. Schopman NCT, Willemsen M, Liu YP, Bradley T, Van Kampen A, Baas F, et al. Deep sequencing of virus-infected cells reveals HIV-encoded small RNAs. Nucleic Acids Res. 2012;40(1):414–27.

41. Nathans R, Chu C-Y, Serquina AK, Lu C-C, Cao H, Rana TM. Cellular microRNA and P bodies modulate host-HIV-1 interactions. Mol Cell. 2009; 34(6):696–709.

42. Ponia SS, Arora S, Kumar B, Banerjea AC. Arginine rich short linear motif of HIV-1 regulatory proteins inhibits dicer dependent RNA interference. Retrovirology. Retrovirology; 2013; 10(1):97.

43. Feinberg MB, Baltimore D, Frankel AD. The role of Tat in the human immunodeficiency virus life cycle indicates a primary effect on transcriptional elongation. Proc Natl Acad Sci U S A. 1991; 88(9):4045–9.

44. du Chéné I, Basyuk E, Lin Y-L, Triboulet R, Knezevich A, Chable-Bessia C, et al. Suv39H1 and HP1gamma are responsible for chromatin-mediated HIV-1 transcriptional silencing and post-integration latency. EMBO J. 2007; 26(2):424–35.

45. Desfosses Y, Solis M, Sun Q, Grandvaux N, Van Lint C, Burny A, et al. Regulation of human immunodeficiency virus type 1 gene expression by clade-specific Tat proteins. J Virol. 2005;79(14):9180–91.

46. Kubota S, Duan L, Furuta RA, Hatanaka M, Pomerantz RJ. Nuclear preservation and cytoplasmic degradation of human immunodeficiency virus type 1 Rev protein. J Virol. 1996;70(2):1282–7.

47. Kewalramani VN, Park CS, Gallombardo PA, Emerman M. Protein stability influences human immunodeficiency virus type 2 Vpr virion incorporation and cell cycle effect. Virology. 1996; 218(2):326–34.

48. Coffin JM, Hughes SH, Varmus HE. Synthesis of Gag and Gag-Pro-Pol Proteins. Cold Spring Harbor Laboratory Press; 1997
