# Supplementary material for: Dynamics of the Gene Regulatory Network of HIV-1 and the Role of Viral Non-coding RNAs on Latency Reversion
Source: Front Physiol. 2018 Sep 28;9:1364. doi: 10.3389/fphys.2018.01364 (PMC6172855; doi:10.3389/fphys.2018.01364)
Supplement: Supplementary file 2 [file Table_2.DOCX]

**Supplementary Table 3. Bifurcation parameters of the ODEs model**

| **Molecule** | **Parameter** | **Reduction (3x)** | **Increase (3x)** | **Biological condition*** |
| --- | --- | --- | --- | --- |
| NF-κB | *k*_0_  *k*_1_  *k*_2_  *k_3_*  *k_4_*  *k_-1_* | Saddle node  **Stable node**  Saddle node  Saddle node  Saddle node  Saddle node | Saddle node  Saddle node  Saddle node  Saddle node  Saddle node  Saddle node | -  Reduction of NF-κB  -  -  -  - |
| HATs | *k_5_*  *k_6_*  *k_7_*  *k_-5_* | Saddle node  Saddle node  Saddle node  Saddle node | Saddle node  Saddle node  Saddle node  Saddle node | -  -  -  - |
| HMTs | *k_8_*  *k_9_*  *k_-8_* | Saddle node  Saddle node  Saddle node | Saddle node  Saddle node  Saddle node | -  -  - |
| p5’LTR | *k_b_*  *k_ac_*  *k_TAR_*  *k_d_*  *k_mt_* | **Stable node**  Saddle node  Saddle node  Saddle node  Saddle node | Saddle node  Saddle node  Saddle node  Saddle node  Saddle node | Inhibition of p5’LTR activity  -  -  -  - |
| p3’LTR | *k_b_*  *k_ac_*  *k_d_*  *k_mt_* | Saddle node  Saddle node  Saddle node  Saddle node | Saddle node  Saddle node  Saddle node  Saddle node | -  -  -  - |
| RNA9kb(N) | *a_1_*  *s_1_*  *τ*  *k_REE_*  *δ_1_* | Saddle node  Saddle node  Saddle node  Saddle node  Saddle node | Saddle node  Saddle node  Saddle node  Saddle node  Saddle node | -  -  -  -  - |
| vsiRNA | *a_2_*  *δ_2_*  *r_1_* | Saddle node  Saddle node  Saddle node | Saddle node  Saddle node  Saddle node | -  -  - |
| vsaRNA | *a_3_*  *δ_3_* | Saddle node  Saddle node | Saddle node  Saddle node | -  - |
| RNA4kb(N) | *s_1_*  *s_2_*  *τ*  *k_REE_*  *δ_5_* | **Stable node**  Saddle node  Saddle node  Saddle node  Saddle node | Saddle node  **Stable node**  **Stable node**  **Stable node**  **Stable node** | Decreases RNA4kb(N)  Decreases RNA4kb(N)  Decreases RNA4kb(N)  Decreases RNA4kb(N)  Decreases RNA4kb(N) |
| RNA2kb(N) | *s_2_*  *k_exp_*  *δ_6_* | **Stable node**  Saddle node  Saddle node | Saddle node  **Stable node**  **Stable node** | Decreases RNA2kb(N)  Decreases RNA2kb(N)  Decreases RNA2kb(N) |
| RNA2kb(C) | *k_exp_*  *r_3_*  *δ_7_* | **Stable node**  Saddle node  Saddle node | Saddle node  **Stable node**  Saddle node | Decreases RNA2kb(C)  Decreases RNA2kb(C)  - |
| RNA4kb(C) | *Τ*  *k_REE_*  *δ_8_*  *r_3_* | Saddle node  Saddle node  Saddle node  Saddle node | Saddle node  Saddle node  Saddle node  Saddle node | -  -  -  - |
| RNA9kb(C) | *Τ*  *k_REE_*  *δ_9_*  *r_3_* | Saddle node  Saddle node  Saddle node  Saddle node | Saddle node  Saddle node  Saddle node  Saddle node | -  -  -  - |
| asRNA | *a_4_*  *r_2_*  *δ_4_* | Saddle node  Saddle node  Saddle node | Saddle node  Saddle node  Saddle node | -  -  - |
| Tat | *a_5_*  *a_6_*  *δ_10_* | Saddle node  Saddle node  Saddle node | Saddle node  Saddle node  Saddle node | -  -  - |
| Rev | *a_7_*  *δ_11_* | Saddle node  Saddle node | Saddle node  Saddle node | -  - |
| Nef | *a_8_*  *δ_12_* | Saddle node  Saddle node | Saddle node  Saddle node | -  - |
| Vpr | *a_9_*  *a_10_*  *δ_13_* | Saddle node  Saddle node  Saddle node | Saddle node  Saddle node  Saddle node | -  -  - |

*These natural situations may change latency stability
